# Model predictive control of consensus-based energy management system for DC microgrid

**DOI:** 10.1371/journal.pone.0278110

**Published:** 2023-01-20

**Authors:** Syed Umaid Ali, Asad Waqar, Muhammad Aamir, Saeed Mian Qaisar, Jamshed Iqbal

**Affiliations:** 1 Department of Electrical Engineering, Center of Excellence in Artificial Intelligence (CoE-AI), Bahria University, Islamabad, Pakistan; 2 Department of Electrical and Computer Engineering, Pak-Austria Fachhochschule Institute of Applied Sciences and Technology, Haripur, Pakistan; 3 Department of Electrical and Computer Engineering, Effat University, Jeddah, Saudi Arabia; 4 Faculty of Science and Engineering, School of Computer Science, University of Hull, Hull, United Kingdom; Wuhan University, CHINA

## Abstract

The increasing deployment and exploitation of distributed renewable energy source (DRES) units and battery energy storage systems (BESS) in DC microgrids lead to a promising research field currently. Individual DRES and BESS controllers can operate as grid-forming (GFM) or grid-feeding (GFE) units independently, depending on the microgrid operational requirements. In standalone mode, at least one controller should operate as a GFM unit. In grid-connected mode, all the controllers may operate as GFE units. This article proposes a consensus-based energy management system based upon Model Predictive Control (MPC) for DRES and BESS individual controllers to operate in both configurations (GFM or GFE). Energy management system determines the mode of power flow based on the amount of generated power, load power, solar irradiance, wind speed, rated power of every DG, and state of charge (SOC) of BESS. Based on selection of power flow mode, the role of DRES and BESS individual controllers to operate as GFM or GFE units, is decided. MPC hybrid cost function with auto-tuning weighing factors will enable DRES and BESS converters to switch between GFM and GFE. In this paper, a single hybrid cost function has been proposed for both GFM and GFE. The performance of the proposed energy management system has been validated on an EU low voltage benchmark DC microgrid by MATLAB/SIMULINK simulation and also compared with Proportional Integral (PI) & Sliding Mode Control (SMC) technique. It has been noted that as compared to PI & SMC, MPC technique exhibits settling time of less than 1μsec and 5% overshoot.

## 1. Introduction

### 1.1 Background

**Fig 1:**
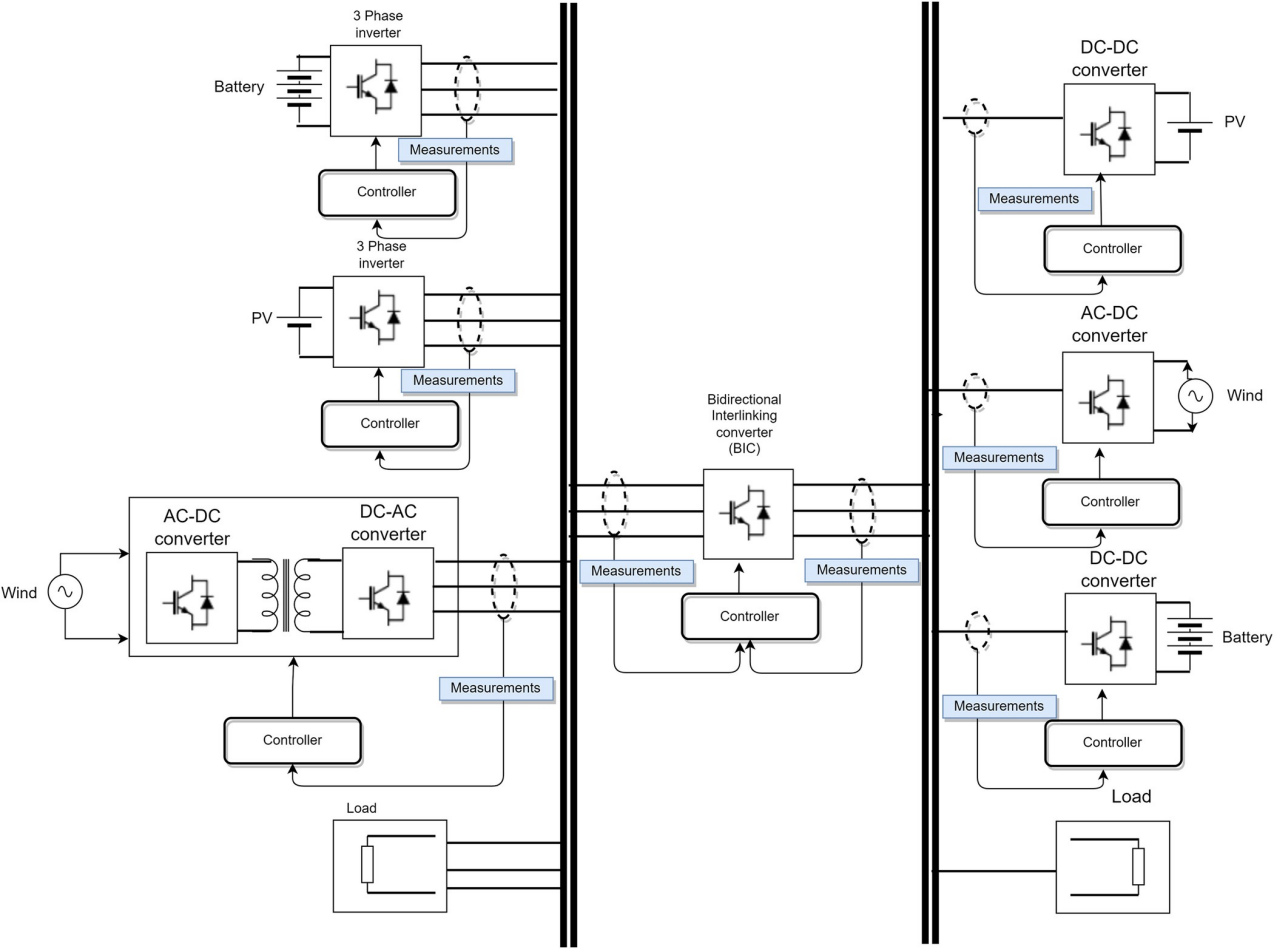
Hybrid microgrid with RES and loads [1].

Microgrids are groups of interconnected generation units and loads that serve a specific area. As depicted in Fig 1 **[1]**, they typically consist of distributed generation (DGs) units, such as wind, solar, and other renewable energy sources, as well as energy storage devices, such as fuel cells, batteries, and supercapacitors. In the DC microgrid (MG), both AC and DC DG units can operate. AC DGs, i.e., wind turbine, etc., can be integrated in DC MG by converting AC output to DC through a rectifier. In DC MG, the main goal is to provide voltage and power regulation for optimal power in the grid **[2]**. GFM converters provide voltage regulation that can be represented as an ideal DC voltage while GFE converters provide power regulation that can be represented as an ideal current source connected to the grid in parallel with high impedance **[3]**.

### 1.2 Challenges

The DC microgrid operation control strategy has been investigated in many studies addressing control aspects, such as DC microgrid voltage regulation [4], different operation modes [5], seamless mode transitions [6], etc. Various energy management systems (EMS) for DC microgrids are analyzed, with each one having different optimization techniques depending on the size of MG and cost optimization [7].

### 1.3 Literature review

An energy management system (EMS) was proposed in [10] for a photovoltaic-based DC microgrid, in which an MPC-based AC/DC converter and PV was used for power regulation, and BESS (BESS and Super Capacitor) used for DC voltage regulation. The roles of power converters are limited. PV is now used only for power regulation. An energy management system was proposed in [8] consisting of two-level control for DC microgrid with PV-based DG, fuel cell, and BESS pack. A BESS pack regulates DC voltage while fuel cell and PV-based DG regulate power. Therefore, again, PV-based DG’s role is fixed, i.e., to regulate power only. An energy management system was proposed in [9] for DC microgrid with PV-based DG and a dual-energy storage system, comprising BESS and super capacitor-based energy storage systems. A dual-energy storage system regulates the DC voltage, and PV-based DG regulates power. Again, PV-based DG’s role is fixed, i.e., to regulate power only. MPC has been used to control voltage and power for demand-side management (DSM) in a micro grid consisting of standalone hybrid renewable energy systems (HRES) [10]. The study considered a DSM capable of rescheduling shift able loads and used Simulink to evaluate the operation of the IMG. During grid-connected mode, power regulation was achieved through direct power MPC (DPMPC), and voltage regulation during islanded mode was achieved through finite control set MPC (FCS-MPC). MPC-PI based control has been used to control voltage and power for a micro grid consisting of PV and hybrid energy storage system (HESS) [11]. Voltage regulation was achieved by SMES through PI-MPC while current regulation is achieved by HESS through FCS-MPC. Again, BESS-based DG’s role is fixed, i.e., to regulate power only. A review paper on MPC was presented that highlighted the contribution of MPC in fault-tolerant control, power quality, and networked micro grids [12]. Frequency regulation in islanded mode has been realized by PID controller in hybrid micro grid with PV, wind and ESS [13]. The controller was tuned by Quasi-oppositional chaotic Selfish-herd optimization (QCSHO) algorithm. MPC has been used to control power for a micro grid consisting of PV and energy storage system (ESS) [14]. PV power is being fed into the load and for charging of ESS. Again, PV-based DG’s role is fixed, i.e., to regulate power only. Power regulation has been realized by classical PID control and state machine control in hybrid micro grid with PV and ESS [15]. PV power is being fed into the load and for charging of ESS. Again, PV-based DG’s role is fixed, i.e., to regulate power only. MPC-PI based control has been used to control voltage and power for a micro grid consisting of PV and hybrid energy storage system (HESS) [16]. Voltage regulation and current regulation was achieved by PI while Super Capacitor (SC) SoC variation is managed by MPC. There are some inherent issues associated with PID such as Pulse Width Modulation (PWM), PID parameters tuning, cascading scheme delayed response issue, complex coordinate transformation, etc. These limitations make PI control implementation too complicated, and it is unable to handle all of the grid’s non-linear complexities. A review paper on energy management solutions with optimization objectives and performance matrices was presented that highlighted the need to improve consumption side energy usage but it lacks in addressing issues concerning to distributed energy resources in new generation smart power grids [17].

### 1.4 Problem statement

Summarizing the literature review for the DC microgrid, the following conclusions can be drawn:

1. DRES are only used as GFE units because of their intermittent nature [8,9]. Therefore, the role of DRES is fixed, i.e., they are used to regulate power only. However, RES at their rated power can be used as GFM DG.
2. In islanded mode, BESS are operated as GFM units to regulate voltage. Charging of BESS is achieved in two stages. In the first stage, charging is achieved on the basis of the difference between generated and load power. Hence, charging current is limited and BESS operates as a GFM unit. In the second stage, as BESS achieves threshold voltage (almost fully charged), its voltage must be kept constant and, for that, BESS should operate as a GFE unit, so that its current begins to taper approaching asymptotically zero, while charging continues. The authors have adopted and refined this idea based on [18].
3. Dual-droop control is used to operate PV and BESS DGs in GFM and GFE modes, with the droop coefficient value chosen for all operating modes having a direct impact on micro grid stability [16]. The proportional integral (PI) control approach was used to provide voltage and power regulation. There are some inherent issues associated with PI such as Pulse Width Modulation (PWM), PID parameter tuning, cascading scheme delayed response issue, complex coordinate transformation, etc. These limitations make PI control implementation too complicated, and it is unable to handle all of the grid’s non-linear complexities.
4. MPC is used to operate PV & HESS in GFE modes [11]. MPC can be used to operate PV in both GFM and GFE modes but it will need to devise multiple control objective in MPC cost function. MPC provides a framework for multiple control objectives in a cost function by associating weighting factor with each objective. It is worth pointing out that, the performance of MPC is deeply influenced by the weighting factors, the tuning of which is still a challenge to be undertaken [19,20].

### 1.5 Scope

**Fig 2:**
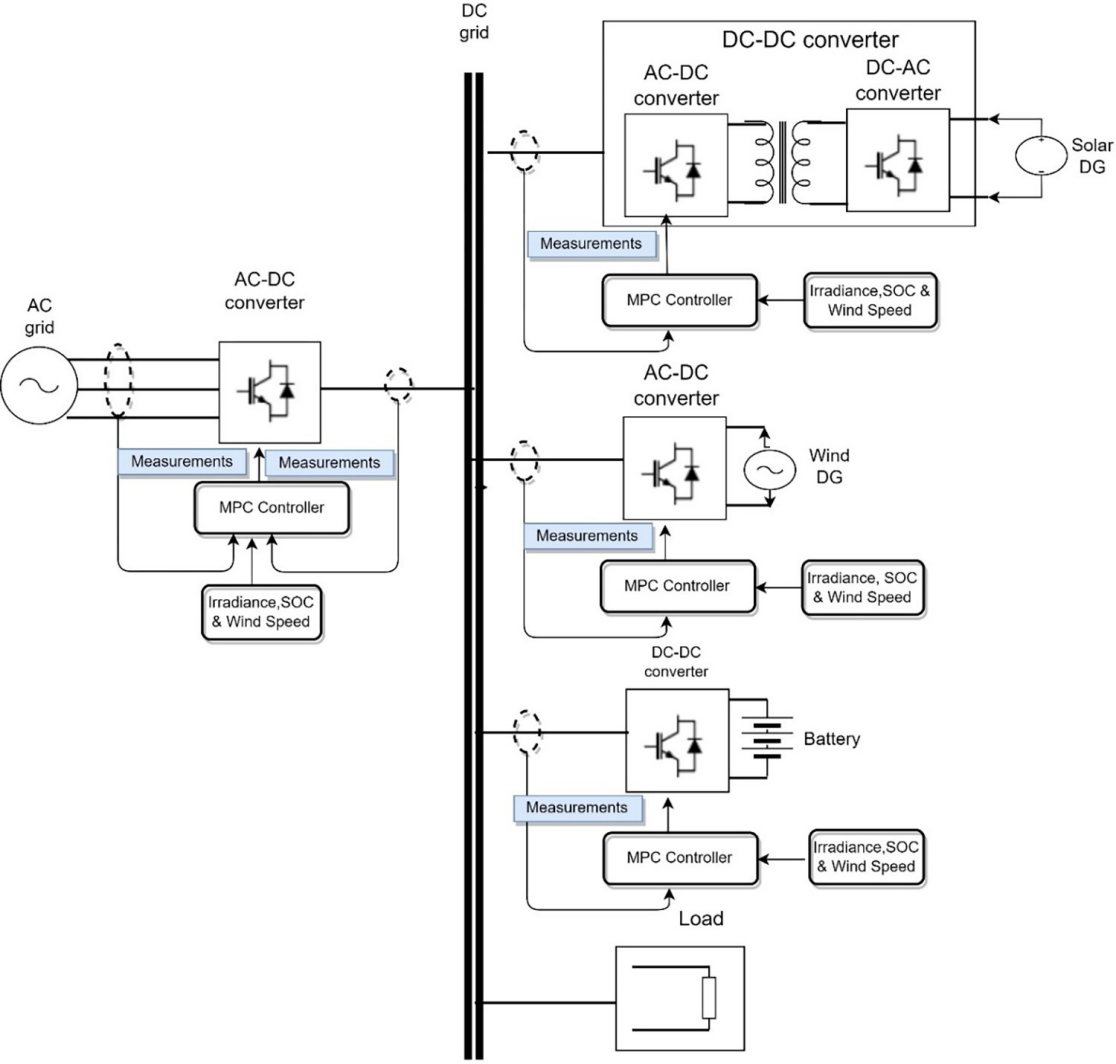
DC microgrid with DRES, BESS, AC/DC converter and load.

In this paper, a consensus-based energy management system (EMS), based on FCS-MPC, is developed for distributed renewable energy sources (DRES) and energy storage system (BESS)-based microgrids. EMS has been proposed to ensure optimal power flow in both grid-connected and standalone operation modes. FCS-MPC has been proposed at the individual converter level for both DRES and BESS with seamless transfer characteristics for operation in both GFM and GFE modes, as explained in Section 2. The EMS power flow mode selection, explained in Section 3, is used to determine the mode of each DRES and BESS’s individual controller. The topology of the microgrid is shown in Fig 2. Here, DRES, such as solar DG and wind DG, are simulated, which is not the main focus of this research. A microgrid consisting of a DC grid with loads and an AC grid has been considered. Solar DG is connected to the DC microgrid through a modular DC/DC PV generation system to provide high-frequency isolation [21]. Wind DG is connected to the DC microgrid through a three-phase two-level voltage source rectifier (VSR). BESS is connected to the DC microgrid through a DC/DC converter. DC and AC microgrids are interconnected through a bidirectional AC/DC interlinking converter with a topology of three-phase two-level voltage source converter (2L-VSC). FCS-MPC-based voltage and power control techniques have been developed for DRES DGs, BESS, and AC/DC converter to regulate voltage by acting as GFM DG, and the current of the DC microgrid by acting as GFE DG. Other practical aspects, such as variable power generation and demand, intermittent power generation, BESS SOC, etc., have been considered. The EMS is developed to ensure stable operation in different operating modes.

### 1.6 Contributions

The main contributions of this work can be detailed as follows:

1. A consensus-based EMS with FCS-MPC is proposed for all the DGs to operate in either GFM or GFE mode in all non-linear conditions by performing multi-variable optimization using auto-tuning weighing factors in the hybrid cost function of MPC. Only one hybrid cost function is used for all the DGs, so transients related to sudden change of cost function do not occur. MPC hybrid cost function with auto-tuning weighing factors will enable every DG to switch between GFM and GFE. This method directly affects the robustness and overall performance of the proposed MPC controller in abnormal or fault conditions. In this method, calculations to select optimal weighing factors are performed in every sampling interval. The weighing factors are calculated dynamically, such that the highest priority is assigned to a given objective which has a large error that should be corrected. So, the weighing factor represents the urgency of correcting the largest error. EMS with FCS-MPC control framework considering fluctuating solar and wind power generation, fluctuating load power demand, and BESS SOC is developed to ensure stable operation and steady transition in both grid-connected and standalone operation modes;
2. Traditionally, as shown in the literature review, DRES are being used as GFE DG in standalone mode due to their intermittent nature. The proposed FCS-MPC allows DRES DGs to act as GFM DG (regulate voltage) when BESS reaches the regulation voltage. DRES will act as GFE DG (regulate power) in grid-connected mode. Thus, MPC-based EMS enables both BESS and DRES to operate in GFM and GFE modes. The proposed MPC scheme achieves better DC grid voltage and power regulation with fewer overshoots and oscillations under fluctuating solar and wind power generation and load consumption profiles;
3. As compared to PI, MPC technique does not require PWM modulators, a PID parameter tuning mechanism, complex coordination transformation, and prior knowledge of variables. In PI, the switching frequency is fixed, while in MPC, it can be variable. In PI, constraint inclusion is not straightforward while in MPC, it can be incorporated.

### 1.7 Organization of paper

The structure of paper is as follows. The DC Microgrid is explained in Section 2. FCS-MPC-based control for DRES (AC/DC converter) and BESS (DC/DC converter) is explained in Section 3. EMS-based power flow modes are explained in in Section 4. Results are discussed in Section 5. Conclusion and future work are discussed in Section 6.

## 2. DC microgrid

A DC microgrid with two DRES, i.e., wind DG and solar DG, and one BESS, i.e., BESS, has been considered in this paper. Fig 2 shows the DC microgrid with AC/DC converter and loads developed as per the EU low voltage benchmark DC microgrid [22]. Three-phase AC/DC converter topology has been used for solar DG, wind DG, and AC/DC converter to transfer power to the DC grid. DC/DC converter has been used for BESS. There are two operation modes, i.e., grid-connected mode and standalone mode, in the proposed MPC control topology for each AC/DC converter and DC/DC converter.

### 2.1. AC/DC converter circuits

2L-VSC topology has been used for AC/DC converter. The MPC weighing factor optimization algorithm runs in discrete-time (DT) domain with fixed sampling interval T_s_. 2L-VSC model equations are in the continuous-time (CT) domain, so sophisticated sampled data models are often needed from CT models.

The CT model of a 2L-VSC has the following equation:

$$\frac{dx(t)}{dt}=\dot{x(t)}=Ax(t)+Bu(t)$$ (1)

where A and B = continuous-time (CT) parameters of the converter, which include DC-link capacitance, load resistance, filter inductance, etc.

x(t) = state variable vector

u(t) = input variable vector

The forward Euler method has been used to find the DT equation for the state Eq in (1). The future sample value (*k* + 1) can be found from the present sample (k) as:

$${\left\{\frac{dx(t)}{dt}\right\}}_{t=k}=\frac{x(k+1)-x(k)}{T_{s}}$$ (2)

where *T*_*s*_ is the step size time.

By substituting (2) into (1), the DT model for the future sample value is obtained:

$$\frac{x(k+1)-x(k)}{T_{S}}=Ax(k)+Bu(k)$$ (3)

### 2.2. AC/DC converter operating modes

The AC/DC converter consists of six IGBT switches, i.e., S1–S6, connected to the DC grid through a 2L-VSC topology with a filter capacitor (C_dc_). The AC/DC converter has two operating modes. Depending on the power output AC source, it can act as GFE DG or GFM DG. Solar DG is connected to the DC microgrid through a modular DC/DC PV generation system to provide high-frequency isolation [21].

### 2.3. Working principle

The switching states of the three-phase AC/DC converter are:

$$S_{a}=\left\{ \begin{array}{l} 1,\;S_{1}\;is\;on\;and\;S_{2}\;is\;off\\ {0,\;S}_{1}\;is\;off\;and\;S_{2}\;is\;on \end{array}\right.$$

$$S_{b}=\left\{ \begin{array}{l} 1,\;S_{3}\;is\;on\;and\;S_{4}\;is\;off\\ {0,\;S}_{3}\;is\;off\;and\;S_{4}\;is\;on \end{array}\right.$$

$$S_{c}=\left\{ \begin{array}{l} 1,\;S_{5}\;is\;on\;and\;S_{6}\;is\;off\\ {0,\;S}_{5}\;is\;off\;and\;S_{6}\;is\;on \end{array}\right.$$

The switching function vector ($\vec{S}$), which combines three-phase representations of switching states, can be expressed as:

$$\vec{S}=\frac{2}{3}(S_{a}+\vec{⍵}S_{b}+{\vec{⍵}}^{2}S_{c})$$ (4)

where $\vec{⍵}=e^{j2\pi /3}=-0.5$ + j0.866 is a vector having 120° phase shift between 3-phases.

## 3. FCS-MPC for DC microgrid

MPC has been considered because it is one of the most promising digital control techniques for power electronics due to its unique ability of combining the discrete nature of the controller with the discrete nature of power converter [23–28] Moreover, MPC is considered as a real and effective solution to traditional controllers based on linear control theory and pulse width modulation [29] and [30].

Table 1 tabulates seven parameters for comparison of MPC with different control techniques [31]. Dynamic response of hysteresis technique is as good as MPC is while MPC is better than hysteresis in terms of constraint inclusion and variable switching frequency. MPC is better than PID based linear control technique as MPC don’t require any modulation stage. MPC is better than PI in terms of constraint inclusion, dynamic response and variable switching frequency. As compared to SMC, MPC has got less control complexity with no modulation stage required. As compared to ANN, constraint inclusion with variable switching frequency is possible in MPC. Very first predictive control technique named as deadbeat predictive control also does not have the ability to perform constraint inclusion as well as variable switching frequency when compared with MPC. So, we can conclude that MPC has got low control complexity, no need of prior knowledge and modulation stage, constraint inclusion feature, excellent dynamic response and controllable switching frequency thus making it an automatic perfect choice to control power converters.

**Table 1:** Comparison of existing techniques with MPC [31].

Parameters	Hysteresis (Relay based)	Linear (PID)	Sliding Mode Control (SMC)	Artificial Neural Network (ANN)	Deadbeat predictive control	MPC
Control Complexity	Low	Medium	high	High	Medium	Low-medium
Model & Parameters	Not Needed	Needed	Needed	Not Needed	Needed	Needed
Prior Knowledge	Not Needed	Not Needed	Not Needed	Needed	Not Needed	Not Needed
Modulation Stage	Not Needed	Needed	Needed	Needed	Needed	Not Needed
Constraint Inclusion	Not Possible	Not Possible	Possible	Not Possible	Not Possible	Possible
Dynamic Response	Excellent	Average	Good	Good	Good	Excellent
Switching Frequency	Variable (Uncontrollable)	Fixed	Fixed	Fixed	Fixed	Variable (Controllable)

### 3.1. AC/DC converter

#### 3.1.1. Current Control (GFE DG)

The space vector equation for output voltage (${\vec{v}}_{conv}$) of AC/DC converter is:

$${\vec{v}}_{conv}=\frac{2}{3}(v_{ao}+\vec{⍵}v_{bo}+{\vec{⍵}}^{2}v_{co})$$ (5)

The relationship between DC bus voltage (*V*_*dc*_) and $\vec{v_{conv}}$, switching function vector ($\vec{S}$) can be defined as:

$${\vec{v}}_{conv}=\vec{S}*V_{dc}.$$ (6)

**Fig 3:**
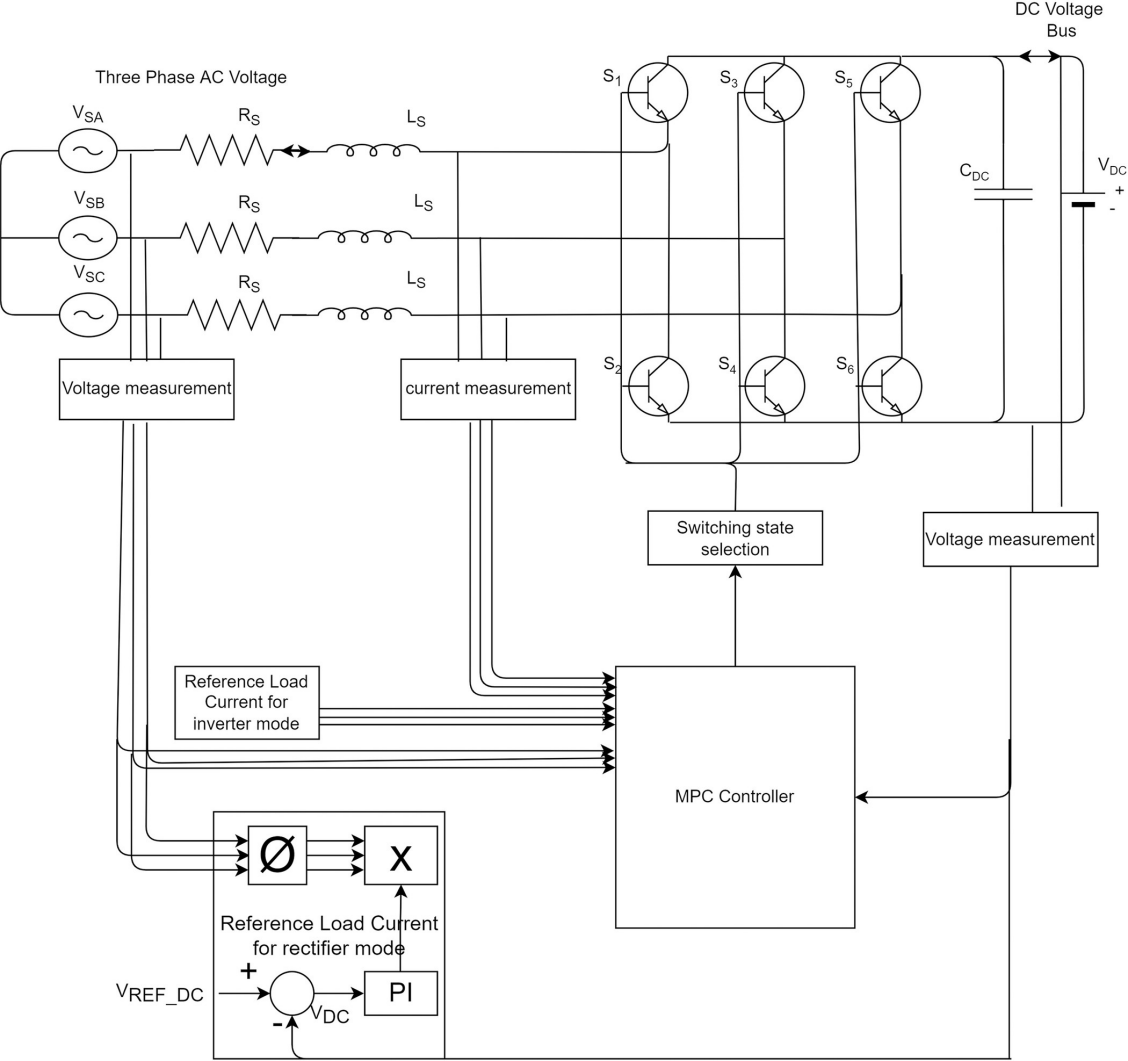
MPC block diagram for GFM and GFE operation through AC/DC converter.

Table 2 lists the possible voltage space vectors for 2L-VSC configuration. Fig 3A shows the GFE DG DC current control algorithm. The AC/DC converter works as a current source rectifier (GFE DG) so that power flows from the AC side to the DC side, as shown in Fig 3. After initialization, reference current calculation is performed by measuring input voltage and current. Then, cost function is calculated for all possible switching states and the switching state with minimum cost function is calculated.

**Table 2:** Voltage switching table.

Switching Combinations			Voltage Vector Outcomes
*S*_*a*_	*S*_*b*_	*S*_*c*_	$\vec{v_{conv}}$
0	0	0	$\vec{v_{1}}$ = 0
0	0	1	$\vec{v_{2}}$ = [−0.33 – j0.577] * V_dc_
0	1	0	$\vec{v_{3}}$ = [−0.33 + j0.577] * V_dc_
0	1	1	$\vec{v_{4}}$ = (−0.67) * V_dc_
1	0	0	$\vec{v_{5}}$ = (0.67) * V_dc_
1	0	1	$\vec{v_{6}}$ = [0.33 − j0.577] * V_dc_
1	1	0	$\vec{v_{7}}$ = [0.33+ j0.577] * V_dc_
1	1	1	$\vec{v_{8}}$ = 0

The relationship between the rectifier input voltage and AC grid output voltage can be written as:

$${\vec{v}}_{s}=L_{s}\frac{d{\vec{i}}_{s\_rec}}{dt}+R_{s}{\vec{i}}_{s\_rec}+\frac{2}{3}(v_{ao}+\vec{⍵}v_{bo}+{\vec{⍵}}^{2}v_{co})-\frac{2}{3}(v_{no}+\vec{⍵}v_{no}+{\vec{⍵}}^{2}v_{no)}$$ (7)

The SVM (space-vector model) of voltage and current is derived as:

$${\vec{v}}_{s}=\frac{2}{3}(v_{sa}+\vec{⍵}v_{sb}+{\vec{⍵}}^{2}v_{sc})$$ (8)

$${\vec{i}}_{s}=\frac{2}{3}(i_{sa}+\vec{⍵}i_{sb}+{\vec{⍵}}^{2}i_{sc})$$ (9)

where *v*_*sc*_, *v*_*sb*_, and *v*_*sa*_ represent three-phase voltages and *i*_*sc*_, *i*_*sb*_, and *i*_*sa*_ represent three-phase currents.


$$\frac{2}{3}(v_{no}+\vec{⍵}v_{no}+{\vec{⍵}}^{2}v_{no})=\frac{2}{3}v_{no}(1+\vec{⍵}+{\vec{⍵}}^{2})=0$$ (10)

Therefore, from Eqs (7)–(9), comprising of rectifier input voltage, the output voltage is:

$${\vec{v}}_{s}=L_{s}\frac{d{\vec{i}}_{s\_rec}}{dt}+R_{s}{\vec{i}}_{s\_rec}+{\vec{v}}_{conv}$$ (11)

Hence, the input current of the AC/DC converter acting as GFE DG [32] is:

$$\frac{d{\vec{i}}_{s\_rec}}{dt}=\frac{1}{L_{s}}\vec{v_{s}}-\frac{R_{s}}{L_{s}}{\vec{i}}_{s\_rec}-\frac{1}{L_{s}}{\vec{v}}_{conv}$$ (12)

#### 3.1.2. Voltage control (GFM DG)

**Fig 4:**
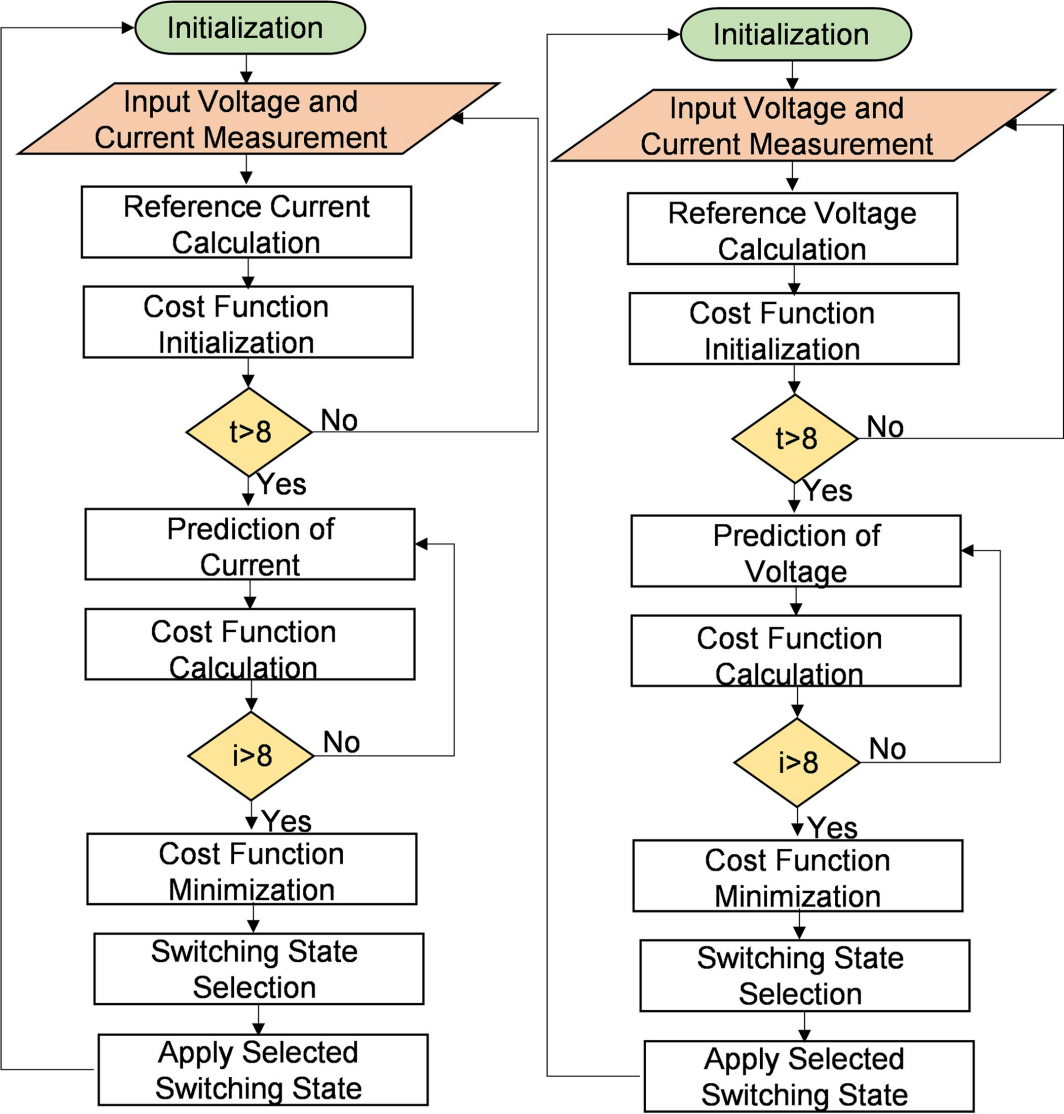
(a) GFE DG control algorithm; (b) GFM DG control algorithm.

The methodology of the MPC algorithm to control DC grid voltage through the AC/DC converter is described in this section. MPC technique operates in the discrete-time domain. Therefore, the time-domain equation of the AC/DC converter represented in (1) is converted into a discrete-time domain. Fig 4B shows the proposed GFM DG control algorithm. After initialization, reference voltage calculation is performed by measuring input voltage and current. The cost function is then calculated for all possible switching states, and the switching state with the minimum cost function is calculated.

Future values of current and voltage are calculated from previous values of (k−1)th sampling interval, using Euler approximation:

$$\frac{dx}{dt}=\frac{\left[x(k)-x(k-1)\right]}{T_{s}}$$ (13)

The future value of current at (k + 1)th sampling instant for AC/DC converter in rectifier mode can be calculated using Euler approximations:

$${\vec{i}}_{s\_rec}(k+1)=\frac{1}{R_{s}T_{s}+L_{s}}\{L_{s}{\vec{i}}_{s\_rec}(k)+T_{s}[v_{s}(k+1)-{\vec{v}}_{conv\_rec}(k+1)]\}$$ (14)

The future value of voltage vector ($\vec{v}(k+1)$) is:

$$\vec{v}(k+1)={\vec{v}}_{conv}(k+1)+ẟ(k+1)$$ (15)

where ẟ(k + 1) = quantization error for voltage vector.


$$\vec{{\vec{i}}_{error}}(k+1)=\vec{i_{s}}(k+1)-{\vec{i}}_{ref\_DC}(k+1)=\frac{1}{R_{s}T_{s}+L_{s}}\{L_{s}{\vec{i}}_{s\_rec}(k)+T_{s}[v_{s}(k+1)-{\vec{v}}_{rec}(k+1)]\}-{\vec{i}}_{ref\_DC}(k+1)$$ (16)

The primary purpose of MPC is to ensure that output current $\left(\vec{i_{s}}\right)$ follows reference current ($\vec{i_{ref}}$), thus, making error current ($\vec{i_{error}}$) zero. This has been achieved using the Lyapunov direct method:

$$L=\frac{1}{2}{\left[{\vec{i}}_{error}(k)\right]}^{T}[{\vec{i}}_{error}(k)]$$ (17)

Hence, the error vector for the input current at future instant ($\vec{{\vec{i}}_{error}}(k+1)$) from (12) is:

The Lyapunov function (rate of change) from (16) and (17) is:

$$\begin{array}{l} {\Delta L}_{rec}(k)=L(\vec{i_{error_{rec}}}(k+1)-L(\vec{i_{error\_rec}}(k)))\\ =\frac{1}{2}{\left[\frac{1}{R_{s}T_{s}+L_{s}}\{L_{s}{\vec{i}}_{s\_rec}(k)+T_{s}[v_{s}(k+1)-{\vec{v}}_{conv_{rec}}(k+1)-\delta (k+1)]-{\vec{i}}_{ref\_DC}(k+1)\}\right]}^{T}X\frac{1}{2}\left[\frac{1}{R_{s}T_{s}+L_{s}}\{L_{s}{\vec{i}}_{s\_rec}(k)+T_{s}[v_{s}(k+1)-{\vec{v}}_{conv\_rec}(k+1)-\delta (k+1)]-{\vec{i}}_{ref\_DC}(k+1)\}\right]-\frac{1}{2}[{{\vec{i}}_{error_{rec}}(k)]}^{T}[{\vec{i}}_{error_{rec}}(k)] \end{array}$$ (18)

The future value of the discrete voltage vector acting as GFM DG [33] is:

$${\vec{v}}_{rec}(k+1)=\frac{L_{s}}{T_{s}}{\vec{i}}_{s\_rec}+{\vec{v}}_{s}(k+1)-\frac{R_{s}T_{s}+L_{s}}{T_{s}}{\vec{i}}_{ref\_DC}(k+1)$$ (19)

### 3.2. DC/DC converter

**Fig 5:**
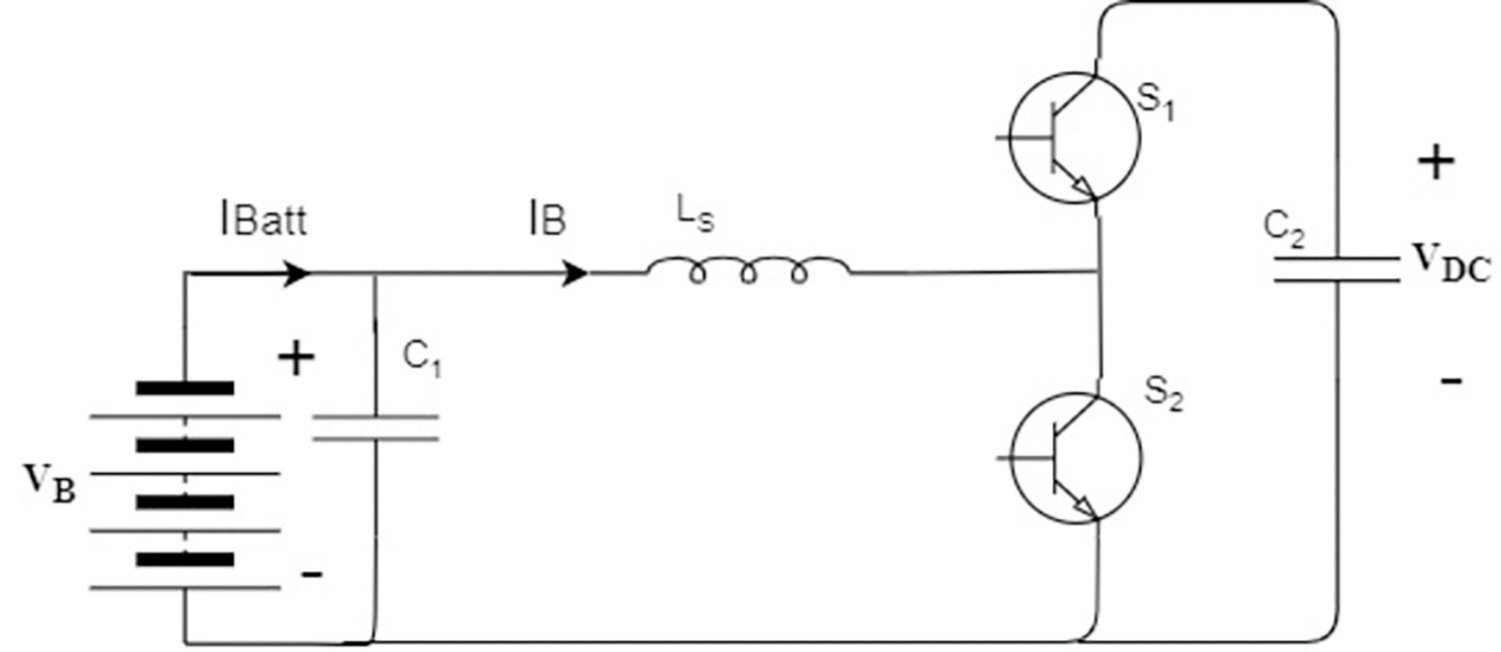
Bidirectional DC/DC converter for BESS.

**Fig 6:**
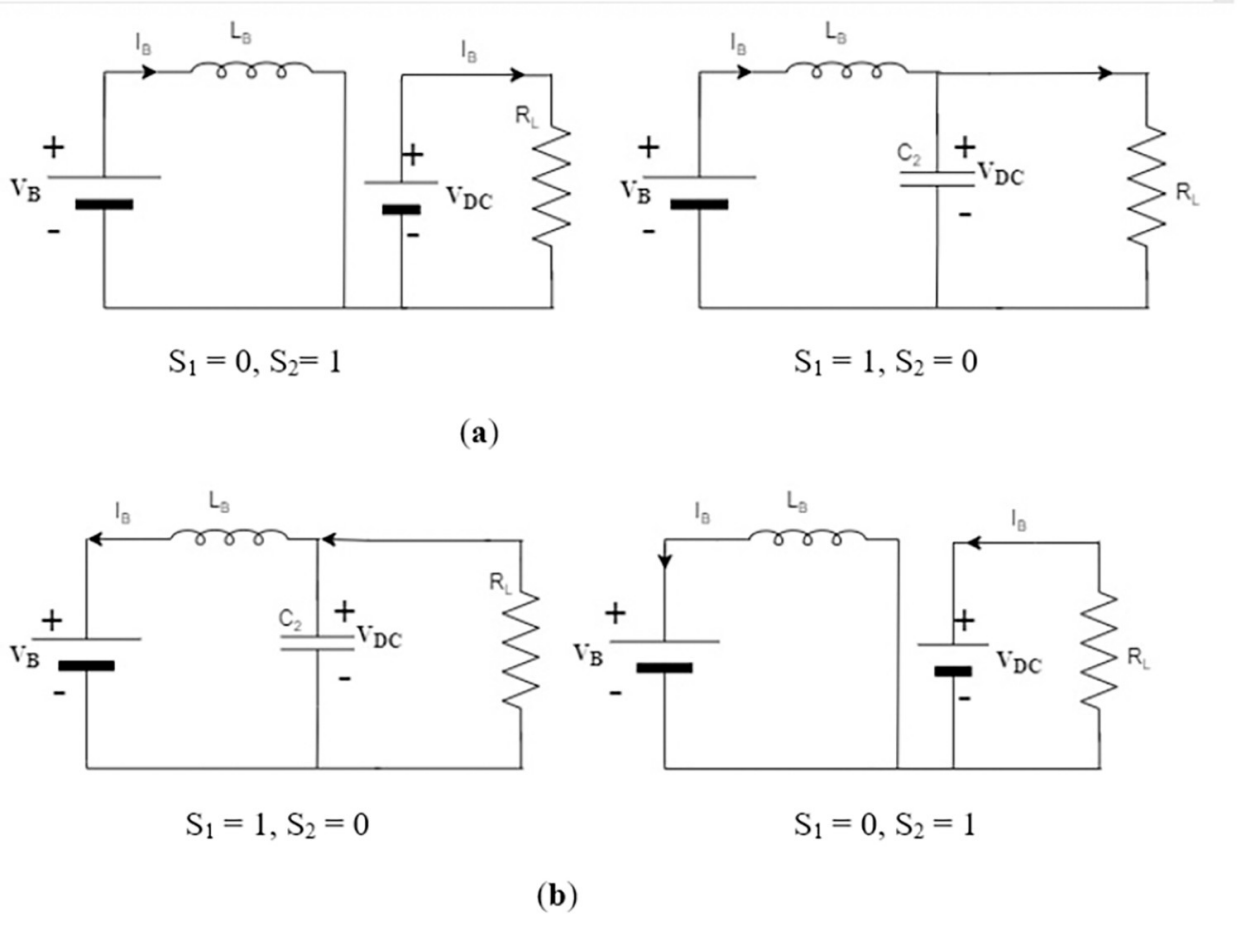
Topologies of bidirectional DC/DC converter (buck-boost) for BESS. (**a**) Boost Mode; (**b**) buck Mode.

The bidirectional buck-boost topology used for DC/DC converter is shown in Fig 5. Buck topology is used for charging the BESS and boost topology is used for discharging the BESS, as shown in Fig 6.

#### 3.2.1. Voltage control (GFM DG)

BESS regulates DC grid voltage through a bidirectional DC/DC converter and will be described in this section.

**Fig 7:**
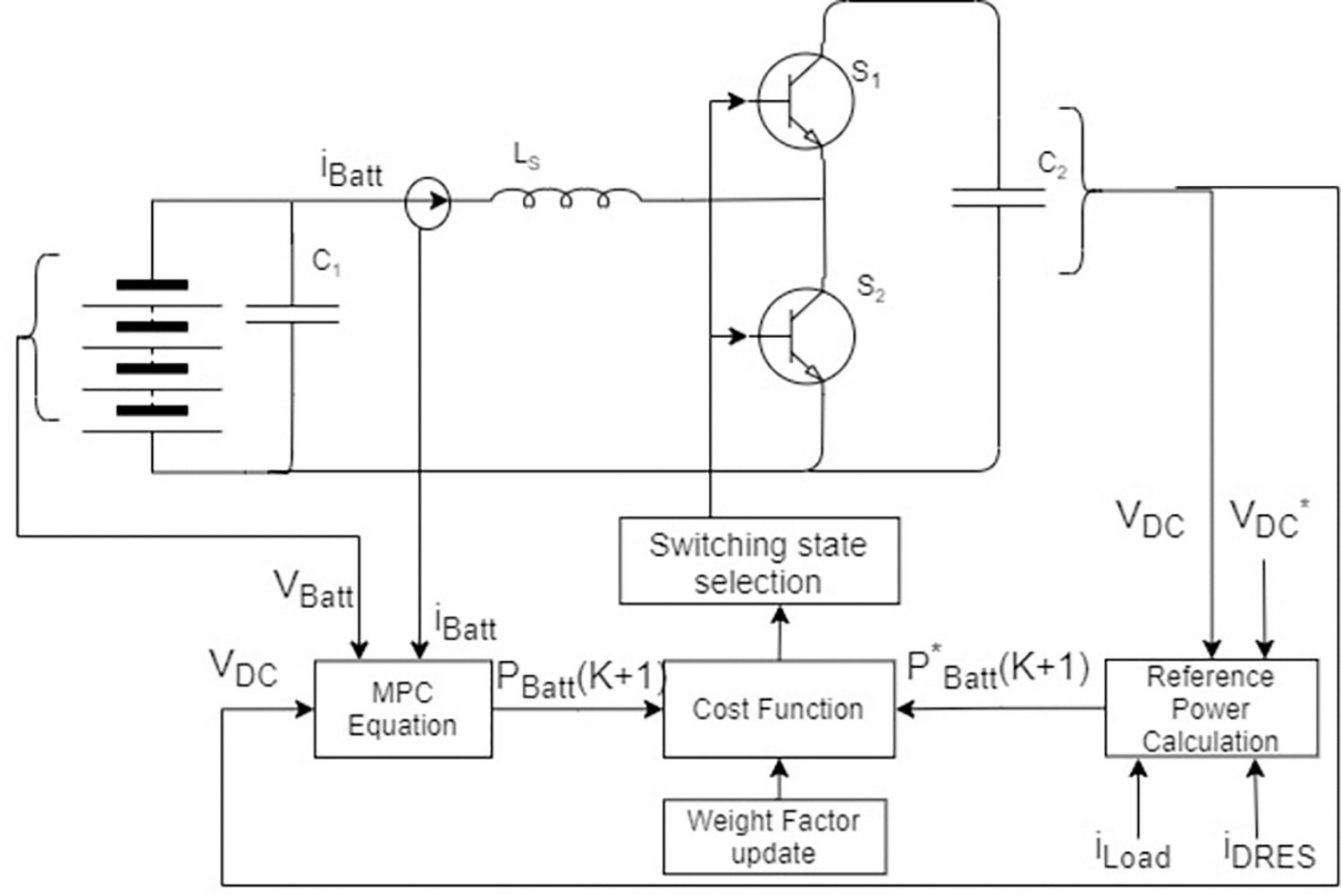
MPC block diagram for GFM operation through DC/DC converter.

DRES output currents, current DC grid voltage, and reference DC grid voltage is used to calculate the required power output required for the BESS converter to regulate the DC grid voltage, as shown in Fig 7. Hence, the cost function for regulating DC grid voltage is:

$$g=|P_{Batt}^{*}(k+1)-P_{Batt}(k+1)|$$ (20)

The reference power output required by BESS converter is:

$$P_{Batt}^{*}(k+1)=|I_{Batt}(k+1).V_{dc}^{*}|$$ (21)

The BESS voltage, current, and DC grid voltage will be used to calculate I_Batt_(k + 1), leading us to calculate P_Batt_(k + 1).


$$V_{dc}(k+1)=V_{dc}(k)+\frac{1}{N}(V_{dc}^{*}-V_{dc}(k))$$ (22)

The Euler approximation equation will be:

$$I_{C}(k+1)=\frac{C_{2}}{T_{S}}(V_{dc}(k+1)-V_{dc}(k))=\frac{C_{2}}{{NT}_{S}}(V_{dc}^{*}-V_{dc}(k))$$ (23)

The BESS current is predicted as:

$$I_{Batt}(k+1)=I_{DRES}(k)-I_{C}(k+1)-I_{load}(k)$$ (24)

Hence, the BESS output power can be predicted as:

$$P_{Batt}(k+1)=|I_{Batt}(k+1).V_{B}(k)|$$ (25)

A cost function is then minimized according to (25).

#### 3.2.2. Current Control (GFE DG)

**Fig 8:**
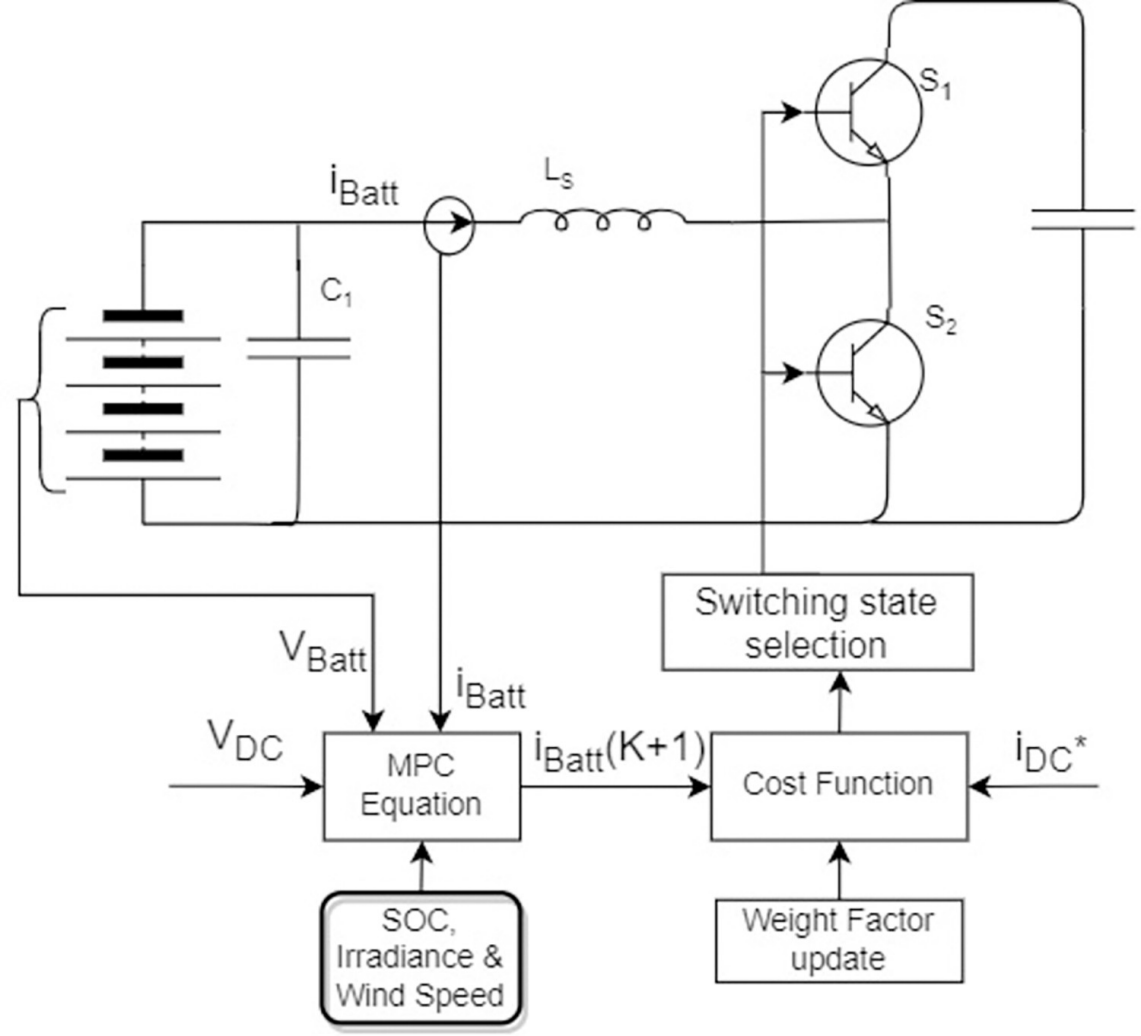
MPC block diagram for GFE operation through DC/DC converter.

BESS regulates DC grid current through MPC, as shown in Fig 8. Two states of switches used in Fig 6 are:

$$\left\{ \begin{array}{c} S_{1}=0,S_{2}=1:\frac{dI_{B}}{dt}L_{B}=V_{B}\\ S_{1}=1,S_{2}=0:\frac{dI_{B}}{dt}L_{B}=V_{B}-V_{DC} \end{array}\right.$$ (26)

Now, discrete-time equation with sampling time can be written as:

$$\left\{ \begin{array}{c} S_{1}=0,S_{2}=1:I_{B}(k+1)=\frac{T_{S}}{L_{B}}V_{B}(k)+I_{B}(k)\\ S_{1}=1,S_{2}=0:I_{B}(k+1)=\frac{T_{S}}{L_{B}}(-V_{DC}(k)+V_{B}(k))+I_{B}(k) \end{array}\right.$$ (27)

The following current-based cost function will be used to control charging or discharging of BESS:

$$g=|I_{refDC}-I_{outDC}|$$ (28)

### 3.3. Hybrid cost function with an auto-tuning weighing factor

MPC hybrid cost function with auto-tuning weighing factors will enable every DG to switch between GFM and GFE. In this paper, a single hybrid cost function has been proposed for both GFM and GFE. The cost functions for solar, wind and BESS with weighing function tuning in different modes are as follows:

$$\min\;g=\{\begin{array}{c} {{ℷ}_{IDC}g_{IDC}+ℷ}_{VDC}g_{VDC}\\ {ℷ}_{IDC}g_{IDC}\\ {ℷ}_{VDC}g_{VDC} \end{array}\vartheta \in \{1-11\}$$ (29)

where cost function is:

$$g_{VDC}=\frac{1}{V_{Rated}}|V_{Ref}(k+1)-V_{Out}(k+1)|$$ (30)

$$g_{IDC}=\frac{1}{I_{Rated}}|I_{Ref}(k+1)-I_{Out}(k+1)|$$ (31)

The above cost function consists of two penalty terms to regulate voltage (V_Out_) and power (I_Out_). The weighing factor (ℷ_VDC_, ℷ_IDC_) is multiplied by the penalty term for prioritizing the multi-objective cost function. The MPC controller auto-tunes the weighing factors, keeping in view the quantized tracking errors of all the variables. The basic aim of this auto-tuning method is to minimize the penalty terms in both grid-connected and standalone modes. The mode detection algorithm is used to detect the mode of operation. The modes of operation are classified as *ϑ* ∈ {1–11}, *ϑ* ∈ {1–6} represents grid-connected mode, and *ϑ* ∈ {7–11} represents standalone mode.

### 3.4. Auto tuning of the weight factors in the hybrid cost function

An auto-tuning method to select the value of the weighing factor (λ_VDC_ and λ_IDC_) is proposed in Fig 6. This method directly affects the robustness and overall performance of the proposed MPC controller in abnormal or fault conditions. In this method, calculations to select optimal weighing factors are performed in every sampling interval.

The weighing factors are calculated dynamically, such that highest priority is assigned to a given objective which has the largest error that should be corrected. So, weighing factor represents the urgency of correcting the largest error.

**Fig 9:**
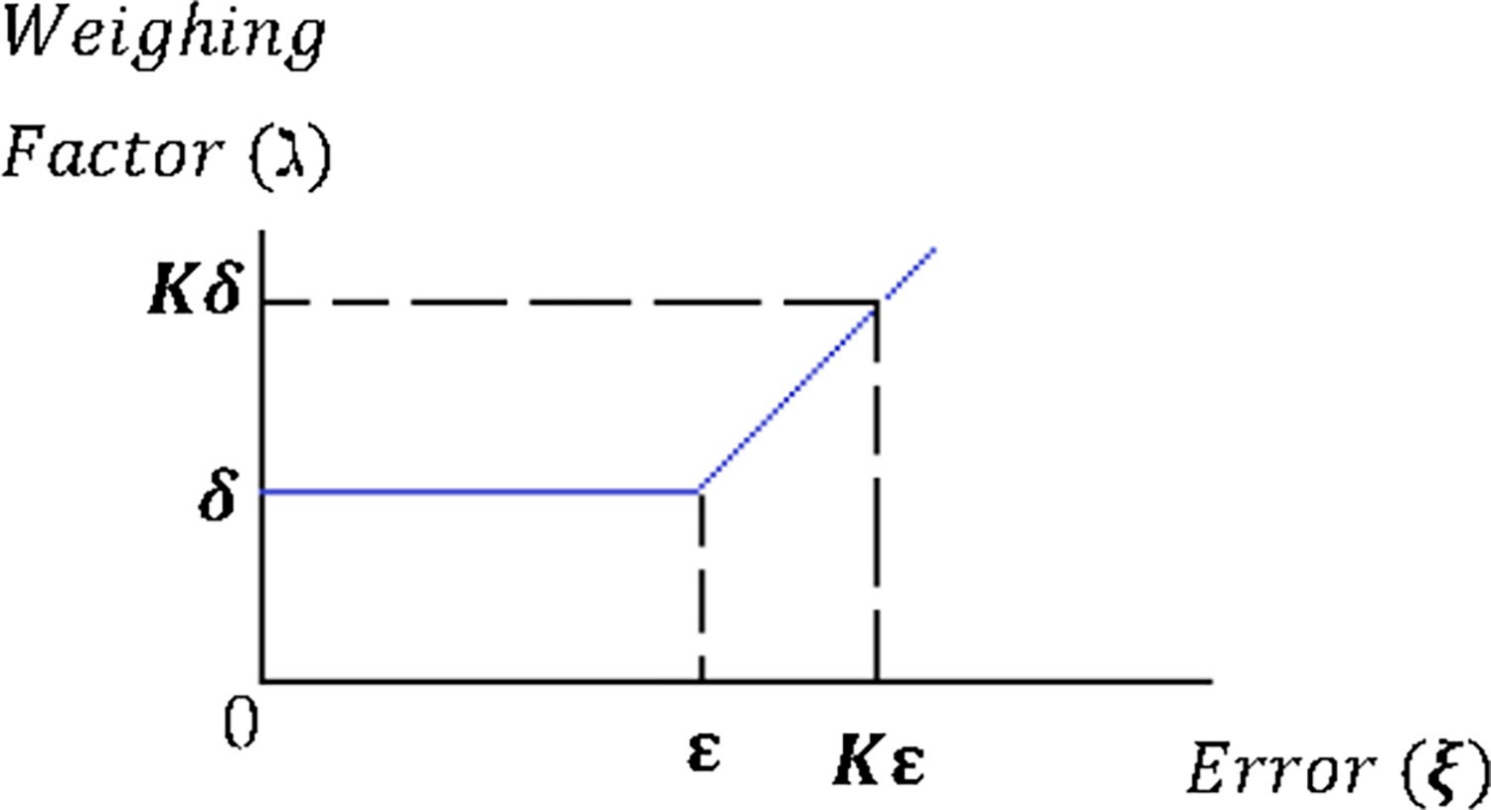
Weighing factor as a function of the error.

In Fig 9, ξ represents the magnitude of the error which is to be corrected by increasing or decreasing converter output. For a large fixed error (Kε), it is essential to apply the appropriate switching state of the converter using MPC to reduce the error. For a small error (ε), an immediate corrective action is not as important; thus, other objectives can be given higher priority. Hence, ξ gives an exact value of the existing error which basically relates the “urgency” of correcting it, so that the weighing factor could be defined proportionally to ξ. Therefore, the weighing factor would be almost zero whenever the error is small, which would increase the error. To avoid this, a minimum value of the weighing factor is taken as one in this paper. This value is used whenever the error is lower than a predefined boundary of ε, as shown in Fig 9. The limit ε represents the maximum admissible error in normal operation. Once the error surpasses ε, the weighing factor increases linearly with the error.

The selection of optimal weighing factors involves predicting errors of all the controllers’ objectives, which are voltage and current in both standalone and grid-connected modes. For every controller, controller objective may vary in both modes, i.e., for AC/DC converter, the controller objective is to predict error for voltage and current in grid-connected mode, for solar, wind and BESS DG’s controller, the objective is to predict error for voltage and power in standalone mode and power in grid-connected mode.

Traditionally, in MPC controllers, the cost function is minimized, and a corresponding switching vector is applied. The proposed algorithm performs auto-tuning of the weighing factor and then minimizes the cost function for the next sampling period. Many of the evaluations for tuning weight factors are based on the computations already conducted. Hence, the cost function is split into two parts with each part affecting individual control objectives:

$$g_{V}=\frac{1}{V_{rated}}|V_{ref}(k+1)-V_{out}(k+1)|\leq {ѱ}_{V}$$ (32)

$$g_{I}=\frac{1}{I_{rated}}|I_{ref}(k+1)-I_{out}(k+1)|\leq {ѱ}_{I}$$ (33)

Ѱ_V_ and Ѱ_I_ are the tracking errors for voltage and current regulation objectives. A minimum value of cost function (g_V_ and g_I_) and their corresponding possible switching states have been selected as follows:

$$\xi_{V}=\min\;g_{V}$$ (34)

$$\xi_{I}=\min\;g_{I}$$ (35)

The above-mentioned minimum values are compared with a small number (ε):

$$\xi_{V}\leq \varepsilon \to {ℷ}_{VDC}=\delta$$ (36)

$$\xi_{I}\leq \varepsilon \to {ℷ}_{IDC}=\delta$$ (37)

**Fig 10:**
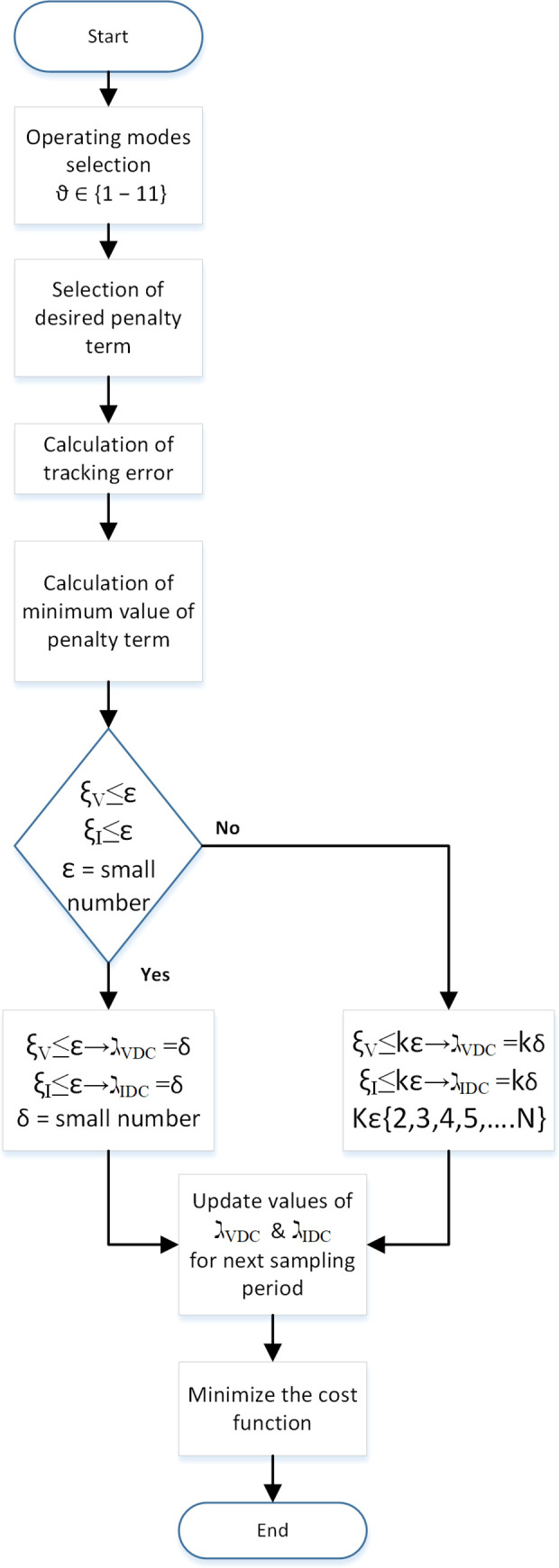
Auto-tuning algorithm of the weight factors in the cost function.

**Fig 11:**
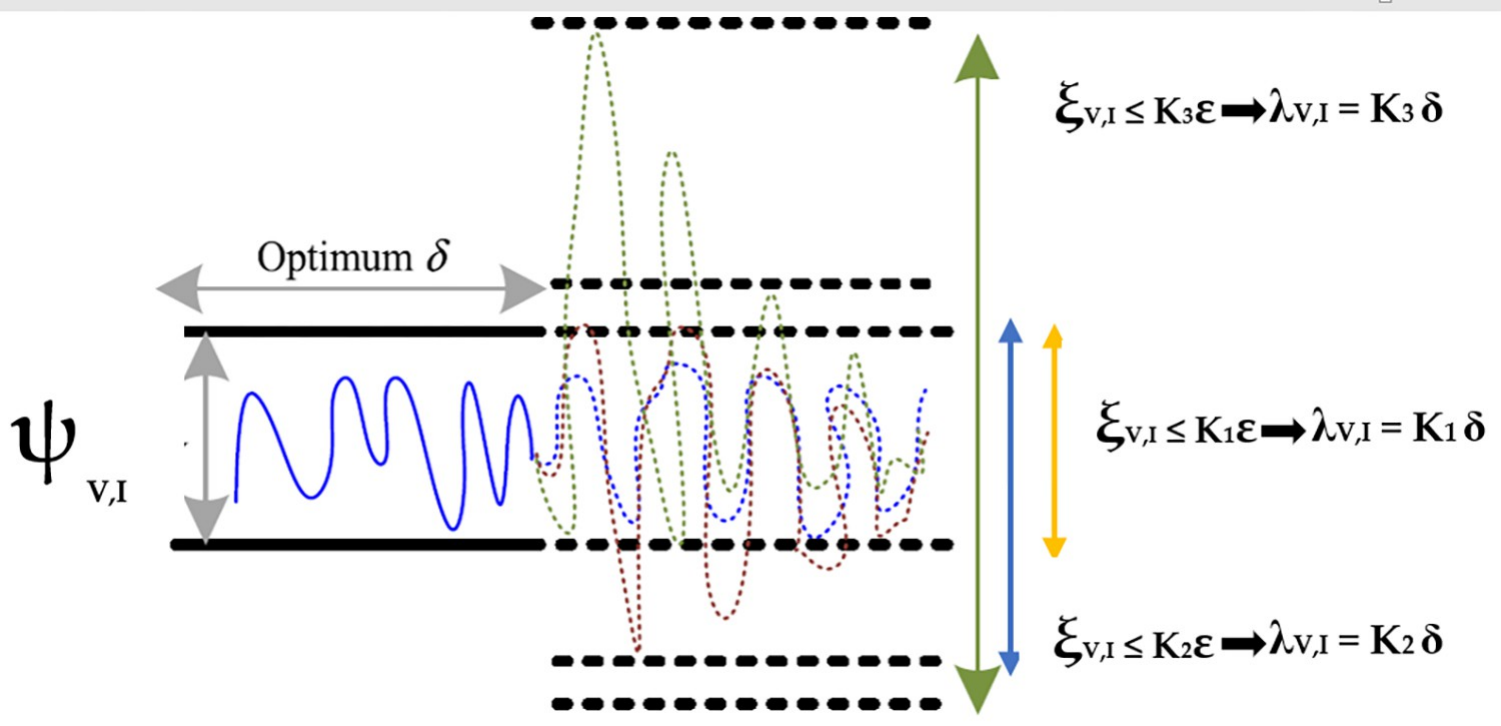
Tuning of weighing factors with respect to tracking error.

The algorithm for weighing factor selection based on absolute errors ξ_V_ and ξ_I_ is shown in Fig 10 [34]. The above equation quantized ξ_V_ and ξ_I_, through which weighing factors are determined by comparing K multiples of ε until Eqs (36) and (37) are satisfied for each variable objective. The corresponding values of weighing factors (ℷ_VDC_ and ℷ_IDC_) are multiplications of K by ε. Three weights, K3, K2 and K1, are shown, with K3 assigned to the variable having more ξ, shown in Fig 11. This algorithm will be run every cycle of sampling time, so weighing factors will be tuned online to minimize the cost function (30) and (31) for the next cycle.

The above equations state that if cost functions (g_V_ and g_I_) are smaller than **ε**, then weighing factors (ℷ_VDC_ and ℷ_IDC_) equal to a sufficiently small number δ is assumed as an initial value. However, if the condition in the above equations is not satisfied, then weighing factors (ℷ_VDC_ and ℷ_IDC_) are assigned higher gain values to the corresponding cost function for minimization in (k + 1) sampling interval, as follows:

$$\xi_{V}\leq K\varepsilon \to {ℷ}_{VDC}=K\delta$$ (38)

$$\xi_{I}\leq K\varepsilon \to {ℷ}_{IDC}=K\delta$$ (39)

where K∈{2,3,….,N}.

**Fig 12:**
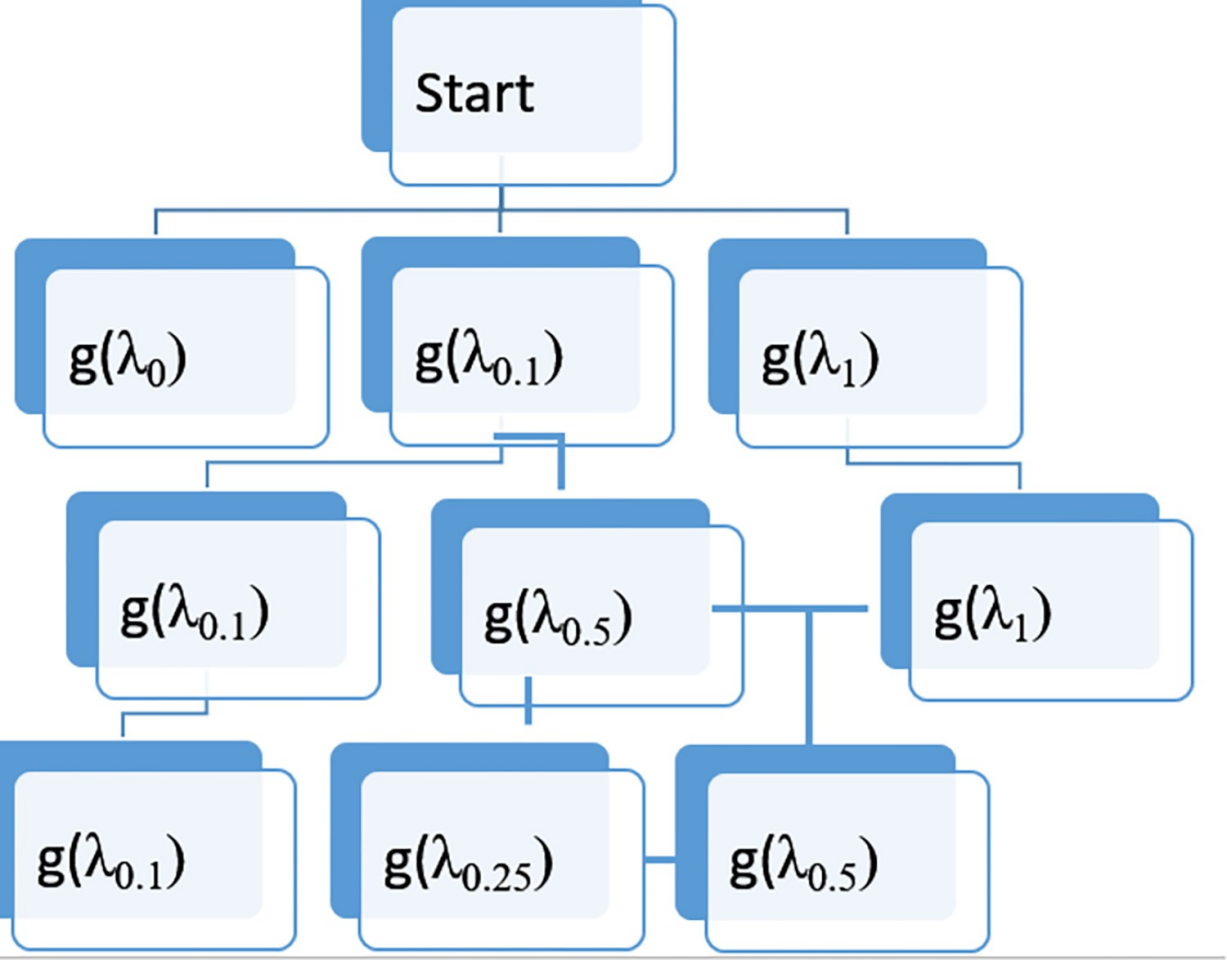
Flow diagram of branch and bound algorithm.

For assigning weighing factors (ℷ_VDC_ and ℷ_IDC_) equal to a sufficiently small number, δ is assumed as an initial value by using the branch and bound algorithm. The algorithm is applied to get the exact value of the weighing factor in one significant figure for which quantized error (*ξ*_*V*_ and *ξ*_*I*_) is equal to zero. In the branch and bound algorithm, exploration of the best possible weighing factor that minimizes the cost function is conducted for different values of “*K*”, and that value of *K* with one significant figure is selected for which quantized error is equal to zero. A flow diagram of the branch and bound algorithm is shown in Fig 12.

## 4. Proposed EMS for DC microgrid

In the grid-connected mode of the proposed DC microgrid, AC power coming through the conventional AC grid is supplied to the DC microgrid through an AC/DC converter. In this mode, the AC/DC converter acts as a GFM DG to regulate DC voltage. Both DRES and BESS DGs act as GFE DG to regulate DC power-sharing.

In standalone mode, the DC microgrid does not get any power from the conventional AC grid. For a reliable operation of a DC microgrid, one DG must assume the role of regulating DC grid voltage by acting as a GFM DG. BESS is charged or discharged, keeping in view the unbalance between generated and consumed power [35]. Therefore, EES acts as GFM DG since BESS current is limited due to power unbalance, while DRES act as GFE DG. As soon as BESS is fully charged and reaches the voltage threshold, BESS voltage should be constant [18]. So, BESS now acts as GFE DG to regulate power, and DRES now acts as GFM DG to regulate voltage. For instance, BESS operates as GFM DG as long as V_batt_ is less than the threshold voltage V_th_. When V_batt_ achieves threshold value, BESS changes its operation mode from GFM to GFE mode. At this moment, DRES acting in GFE mode should change its mode to GFM mode to regulate DC microgrid voltage. Thus, DRES continue acting as GFM DG as long as it has enough power to supply power to the DC microgrid. Otherwise, BESS DG reassumes the role of GFM DG. If BESS does not have enough power, then load shedding will be conducted. So, every DRES and BESS DG has one MPC hybrid cost function with an auto-tuning weighing factor for switching between GFM and GFE control modes.

### 4.1. Operation of proposed EMS for DC microgrid

In our proposed EMS, the DC microgrid operating mode will be determined based on generated and load powers of DRES, BESS, and AC/DC converter.

The power flow of the DC microgrid is controlled by calculating the load and generated power of DRES. The generated power is calculated from solar and wind DRES, respectively, as shown below:

$$P_{Gen}=P_{Solar}+P_{Wind}$$ (40)

$$P_{Load}=P_{Load}$$ (41)

After receiving generated power and load power values from respective DRES, the grid-connected mode will be classified for Grid_status_ = 1 and standalone mode for Grid_status_ = 0.

### 4.2. Grid-connected mode

**Fig 13:**
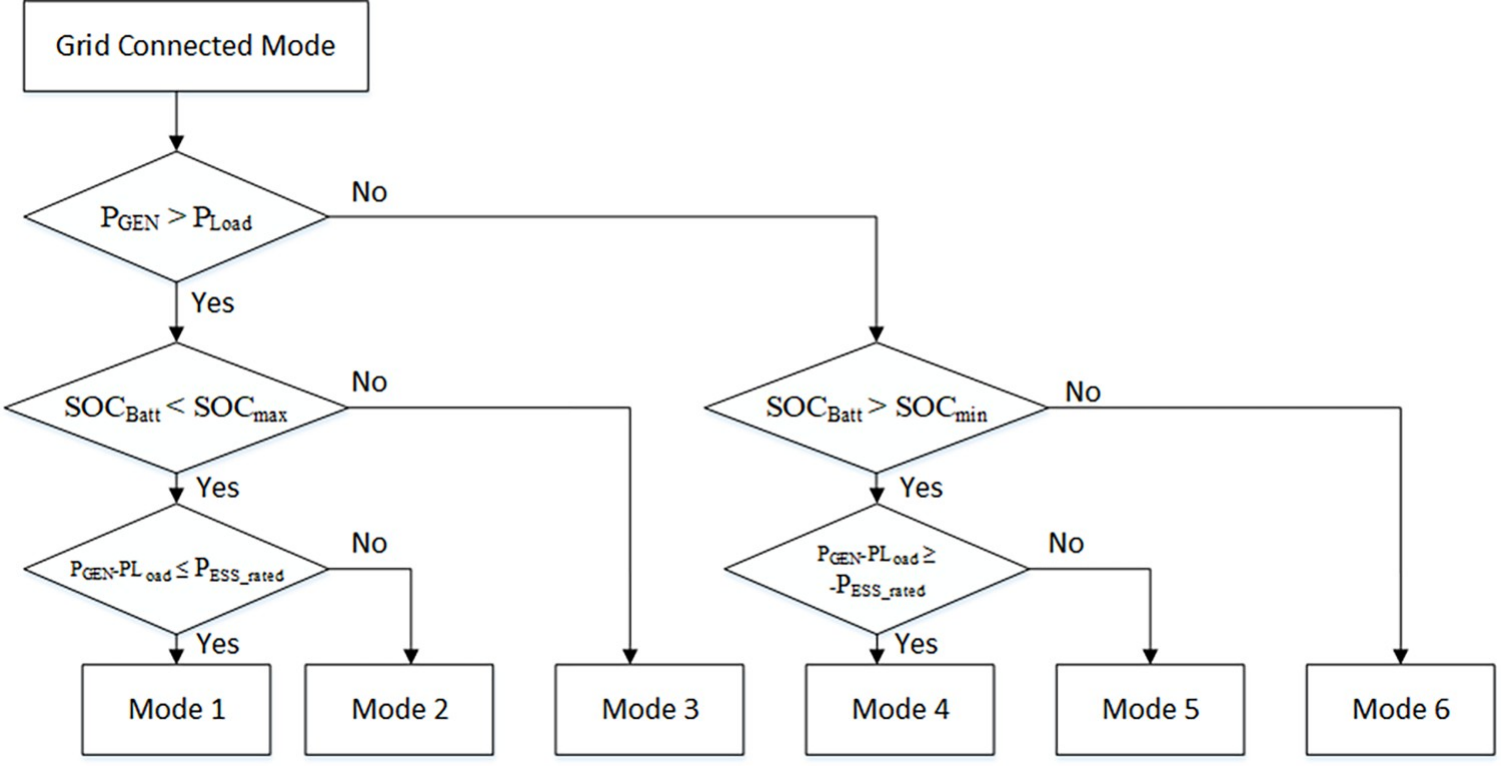
Flow chart of EMS for DC microgrid in grid-connected mode.

Fig 13 shows the flow chart of EMS for the DC microgrid in grid-connected mode. In this mode, the generated power is compared with the load power. If generated power is greater than load power (P_Gen_ > P_load_), then either Mode 1, 2 (surplus power) or Mode 3 (charging) will be chosen, depending on BESS SOC, rated power of BESS converter (P_BESS_rated_), and amount of available charging power (P_Gen_-P_Load_). Here, the interval of ±0.1 is used to avoid unnecessary chattering.

#### 4.2.1. Mode 1

If power is surplus and BESS SOC is less than the maximum allowable SOC, EMS will determine that BESS needs charging, so mode 1 will be adopted. Surplus power (P_SURPLUS_) is determined by comparing the difference between generated and load power (P_GEN_ − P_Load_):

$$P_{Surplus}=P_{Gen}-P_{Load}$$ (42)

Surplus power must be less than or equal to the rated power of the BESS:

$$P_{Surplus}\leq P_{ESS\_rated}$$ (43)

The amount of charging power is calculated by the equation:

$$P_{Char}=P_{Gen}-P_{Load}={[P}_{Solar}+P_{Wind}-P_{Load}]>0$$ (44)

In this mode, the AC/DC converter is regulating voltage, DRES is regulating power, and BESS is in charging mode, so cost functions for all converters will be:

$$\begin{array}{c} {AC/DC\;converter=ℷ}_{VDC}{\left(V_{refDC}-V_{outDC}\right)}^{2}\\ Solar={ℷ}_{IDC}{\left(I_{refDC}-I_{outDC}\right)}^{2}\\ Wind={ℷ}_{IDC}{\left(I_{refDC}-I_{outDC}\right)}^{2}\\ Battery={ℷ}_{IDC}{\left(I_{refDC}-I_{outDC}\right)}^{2} \end{array}$$ (45)

#### 4.2.2. Mode 2

If surplus power is more than the rated power of the BESS, then EMS will operate the DC microgrid in mode 2:

$$P_{Surplus}\geq P_{ESS\_rated}$$ (46)

Hence, the BESS charging current must be limited, and the remaining surplus power flows to the AC microgrid. Therefore, BESS power will be:

$${P_{Batt}=P}_{ESS\_rated}$$ (47)

The remaining surplus power flows to the AC microgrid through the AC/DC converter. Additionally, the AC/DC converter performs voltage regulation of the DC microgrid as well:

$$P_{AC/DC\;converter}=P_{Gen}-P_{Load}-P_{ESS\_rated}>0$$ (48)

In this mode, the AC/DC converter is regulating voltage and exporting power to the AC microgrid as well, DRES is regulating power, and BESS is in charging mode, so cost functions for all converters will be:

$$\begin{array}{c} {AC/DC\;converter=ℷ}_{VDC}{\left(V_{refDC}-V_{outDC}\right)}^{2}+{(-ℷ}_{IAC}{\left(I_{refAC}-I_{outAC}\right)}^{2})\\ Solar={ℷ}_{IDC}{\left(I_{refDC}-I_{outDC}\right)}^{2}\\ Wind={ℷ}_{IDC}{\left(I_{refDC}-I_{outDC}\right)}^{2}\\ Battery={ℷ}_{IDC}{\left(I_{refDC}-I_{outDC}\right)}^{2} \end{array}$$ (49)

#### 4.2.3. Mode 3

If BESS SOC is greater than maximum SOC, no charging will be required. So, EMS will operate in this mode, and all the surplus power will flow to the AC grid through the AC/DC converter:

$$P_{AC/DCconverter}=P_{Gen}-P_{Load}>0$$ (50)

Therefore, when generated power is greater than load power, Mode 1, 2, or 3 will be selected. The AC/DC converter regulates voltage and exports surplus power to the AC microgrid. DRES and BESS are regulating power, so cost functions for all converters will be:

$$\begin{array}{c} {AC/DC\;converter=ℷ}_{VDC}{\left(V_{refDC}-V_{outDC}\right)}^{2}+{(-ℷ}_{IAC}{\left(I_{refAC}-I_{outAC}\right)}^{2})\\ Solar={ℷ}_{IDC}{\left(I_{refDC}-I_{outDC}\right)}^{2}\\ Wind={ℷ}_{IDC}{\left(I_{refDC}-I_{outDC}\right)}^{2}\\ Battery={-ℷ}_{IDC}{\left(I_{refDC}-I_{outDC}\right)}^{2} \end{array}$$ (51)

#### 4.2.4. Mode 4

When generated power is less than load power, the BESS is discharged to fulfil the load power requirement, provided BESS SOC is greater than the minimum threshold SOC. Then, required discharge power is compared with the BESS converter rated capacity (P_BESS_rated_). If the required discharge power is less than P_BESS_rated_, then mode 5 is adopted by EMS. In this mode BESS discharges the power to fulfil the load requirement:

$$P_{ESS}=P_{Gen}-P_{Load}<0$$ (52)

In this mode, the AC/DC converter is regulating voltage, DRES and BESS are regulating power, so cost functions for all converters will be:

$$\begin{array}{c} {AC/DC\;converter=ℷ}_{VDC}{\left(V_{refDC}-V_{outDC}\right)}^{2}\\ Solar={ℷ}_{IDC}{\left(I_{refDC}-I_{outDC}\right)}^{2}\\ Wind={ℷ}_{IDC}{\left(I_{refDC}-I_{outDC}\right)}^{2}\\ Battery={-ℷ}_{IDC}{\left(I_{refDC}-I_{outDC}\right)}^{2} \end{array}$$ (53)

#### 4.2.5. Mode 5

If the required discharge power is greater than P_BESS_rated_, then mode 5 is adopted by EMS. In this mode, the discharge power by the BESS will be:

$$P_{ESS}=-P_{ESS\_rated}$$ (54)

Additional required power will be imported from the AC microgrid through the AC/DC converter:

$$P_{AC/DC\;converter}=P_{Gen}-P_{Load}-P_{ESS_{rated}}<0$$ (55)

In this mode, the AC/DC converter regulates voltage and imports required power from the AC microgrid. DRES and BESS are regulating power, so cost functions for all converters will be:

$$\begin{array}{c} {AC/DC\;converter=ℷ}_{VDC}{\left(V_{refDC}-V_{outDC}\right)}^{2}+{(ℷ}_{IAC}{\left(I_{refAC}-I_{outAC}\right)}^{2}\\ Solar={ℷ}_{IDC}{\left(I_{refDC}-I_{outDC}\right)}^{2}\\ Wind={ℷ}_{IDC}{\left(I_{refDC}-I_{outDC}\right)}^{2}\\ Battery={-ℷ}_{IDC}{\left(I_{refDC}-I_{outDC}\right)}^{2} \end{array}$$ (56)

#### 4.2.6. Mode 6

If BESS SOC is at the minimum level, then discharge through the BESS is not possible and mode 6 is adopted by EMS, in which required power is imported from the AC microgrid through the AC/DC converter:

$$P_{AC/DCconverter}=P_{Gen}-P_{Load}<0$$ (57)

In this mode, the AC/DC converter regulates voltage and imports required power from the AC microgrid. DRES is regulating power, so cost functions for all converters will be:

$$\begin{array}{c} {AC/DC\;converter=ℷ}_{VDC}{\left(V_{refDC}-V_{outDC}\right)}^{2}+{(ℷ}_{IAC}{\left(I_{refAC}-I_{outAC}\right)}^{2}\\ Solar={ℷ}_{IDC}{\left(I_{refDC}-I_{outDC}\right)}^{2}\\ Wind={ℷ}_{IDC}{\left(I_{refDC}-I_{outDC}\right)}^{2}\\ Battery=0 \end{array}$$ (58)

Therefore, in grid-connected mode, when DRES-generated power is less than load power, EMS will select mode 5 to let the BESS discharge the required power to fulfil the load power. If BESS-discharged power is not enough to fulfil all the load power requirements, then mode 4 is selected to let the BESS discharge and import the remaining required power from the AC grid. If BESS discharge power is not possible, mode 6 is selected to import power from the AC grid.

### 4.3. Standalone mode

**Fig 14:**
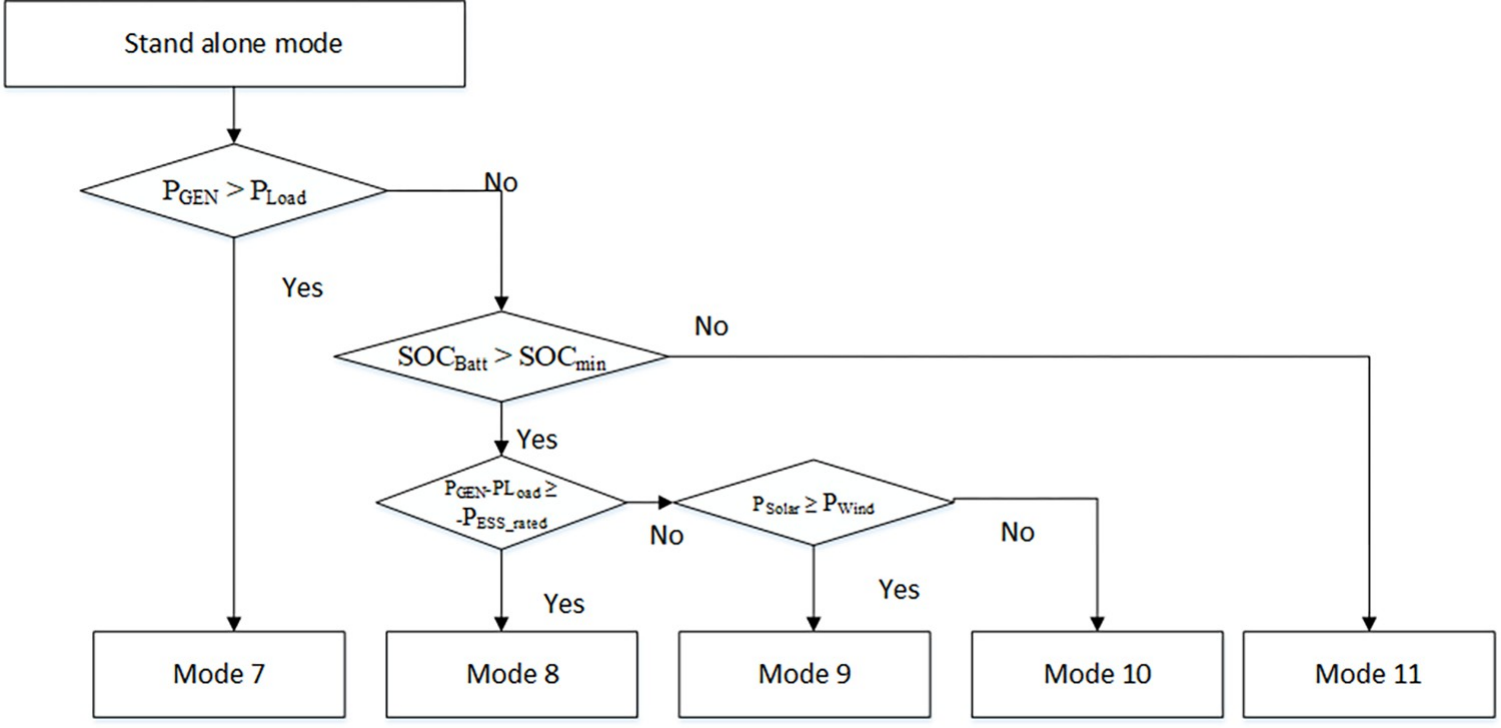
Flow chart of EMS for DC microgrid in standalone mode.

Fig 14 shows the flow chart of EMS for the DC microgrid in standalone mode. The DC microgrid is disconnected from the AC microgrid due to a fault in this mode. Therefore, a Grid_status_ = 0 signal is generated to let DRES and BESS know that the DC microgrid is in standalone mode. DRES and BESS regulate voltage and power-sharing depending upon BESS SOC and voltage. Here, the interval of ±0.1 is used to avoid unnecessary chattering.

#### 4.3.1. Mode 7

In this mode, generated power (P_Gen_) is greater than load power (P_Load_) so the BESS is being charged as its being implemented by EMS in mode 3.

In standalone mode, voltage regulation is to be conducted either by DRES or BESS, depending upon BESS voltage. If the BESS is fully charged and reaches its voltage threshold, BESS voltage should be kept constant [23]. The BESS will now act as GFE DG to regulate power, and DRES will act as GFM DG to regulate voltage. The BESS will operate as GFM DG in this mode as long as V_batt_ is less than the threshold voltage Vth.


$$\begin{array}{c} AC/DC\;converter=0\\ Solar={ℷ}_{IDC}{\left(I_{refDC}-I_{outDC}\right)}^{2}\\ Wind={ℷ}_{IDC}{\left(I_{refDC}-I_{outDC}\right)}^{2}\\ Battery={ℷ}_{VDC}{\left(V_{refDC}-V_{outDC}\right)}^{2} \end{array}$$ (59)

#### 4.3.2. Mode 8

When V_batt_ achieves threshold value, the BESS changes its operation mode from GFM to GFE mode. At this moment, DRES acting in GFE mode should change its mode to GFM mode to regulate DC microgrid voltage. Thus, DRES will continue acting as GFM DG as long as it has enough power to supply the DC microgrid. Wind DG produces more power than solar DG and acts as GFM DG, with solar DG acting as GFE DG. Hence, cost functions for all converters will be:

$$\begin{array}{c} AC/DC\;converter=0\\ Solar={ℷ}_{IDC}{\left(I_{refDC}-I_{outDC}\right)}^{2}\\ Wind={ℷ}_{VDC}{\left(V_{refDC}-V_{outDC}\right)}^{2}\\ Battery={ℷ}_{IDC}{\left(I_{refDC}-I_{outDC}\right)}^{2} \end{array}$$ (60)

#### 4.3.3. Mode 9

Solar DG produces more power than wind DG in this mode, so it acts as GFM DG while wind DG acts as GFE DG. Thus, cost functions for all converters will be:

$$\begin{array}{c} AC/DC\;converter=0\\ Solar={ℷ}_{VDC}{\left(V_{refDC}-V_{outDC}\right)}^{2}\\ Wind={ℷ}_{IDC}{\left(I_{refDC}-I_{outDC}\right)}^{2}\\ Battery={ℷ}_{IDC}{\left(I_{refDC}-I_{outDC}\right)}^{2} \end{array}$$ (61)

#### 4.3.4. Mode 10

In this mode, generated power (P_GEN_) is less than load power (P_Load_), so the BESS is discharged equivalent to the BESS discharge rated power P_BESS_rated_ to provide required load power. BESS acts as GFM DG to regulate voltage, and DRES act as GFE DG to regulate power. Thus, cost functions for all converters will be same as (59).

#### 4.3.5. Mode 11

When the generated power (P_GEN_) is less than load power (P_Load_), and BESS SOC is less than the minimum threshold SOC, there is no choice for EMS except to operate in load constraint mode in which output load power is set to zero and DC microgrid operation is stopped. Table 3 summarized the roles of power converters in all the modes.

**Table 3:** Roles of power converters in all cases.

Mode	Solar DG	Wind DG	BESS DG	AC/DC Converter
1	GFE	GFE	GFE (Charging)	GFM (DC Grid)
2	GFE	GFE	GFE (Charging)	GFM (DC Grid)
3	GFE	GFE	Idle	GFM (DC Grid)
4	GFE	GFE	GFE (Discharging)	GFM (DC Grid)
5	GFE	GFE	GFE (Discharging)	GFM (DC Grid)
6	GFE	GFE	Idle (low SOC)	GFM (DC Grid)
7	GFE	GFE	GFM (Charging)	GFM (AC Grid)
8	GFE	GFM	GFE (Charging)	GFM (AC Grid)
9	GFM	GFE	GFE (Charging)	GFM (AC Grid)
10	GFE	GFE	GFM (Discharging)	GFM (AC Grid)
11	Idle	Idle	Idle	Idle

### 4.4 Sensitivity analysis of the controller

#### Sensitivity analysis of AC-DC converter

**Fig 15:**
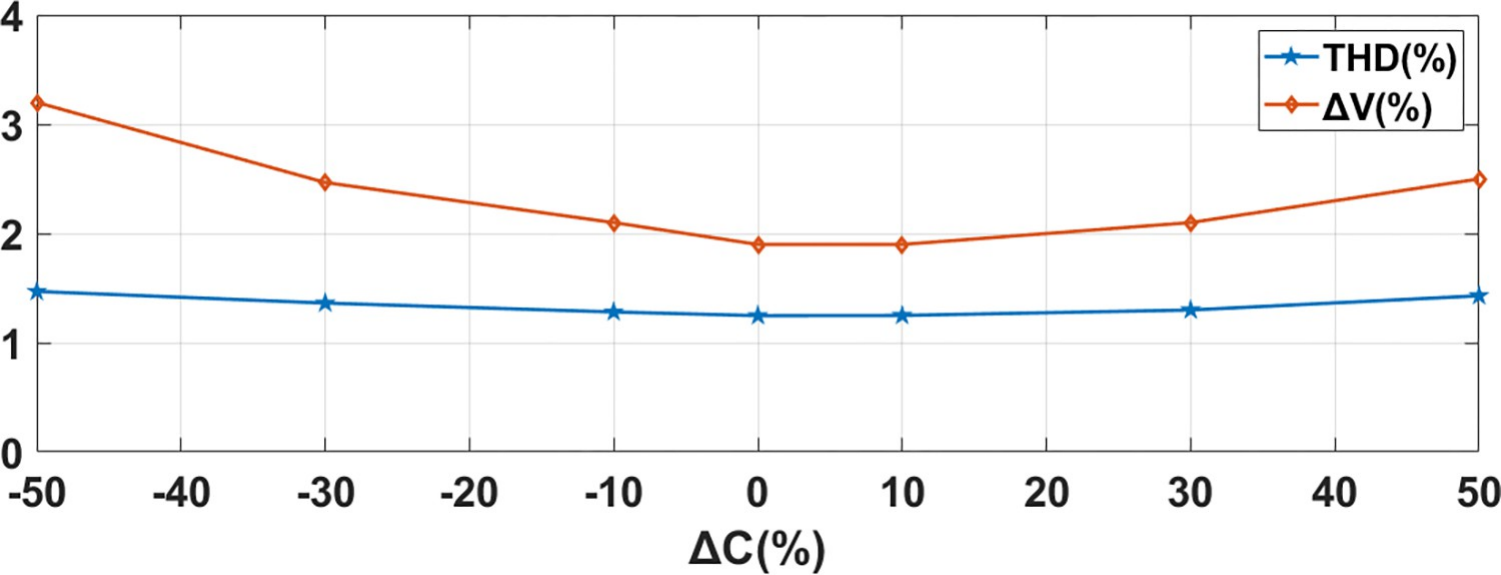
Sensitivity of proposed MPC controller to variations in capacitor value for AC-DC Converter.

**Fig 16:**
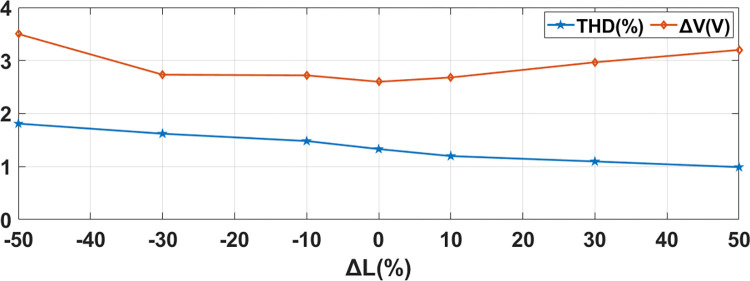
Sensitivity of proposed MPC controller to variations in inductor value for AC-DC Converter.

Sensitivity Analysis on AC-DC converter has been done with respect to the variation of parameters (Filtering inductor (L_s_) and Capacitor (C_2_)) on system response. Parameters have been changed from -50% to +50% of their values. Seven testing points are taken into consideration. For simplicity one parameter will be varied at a time. Performance parameters of Capacitor Voltage (V) and Grid Current THD have been chosen. Practically variation in L and C are within ±10% range. As shown in Figs 15 and 16, these variations do not have considerable effect on capacitor voltage and grid current THD. These results show that proposed MPC algorithm is less sensitive to parameter change.

#### Sensitivity analysis of DC-DC converter

Sensitivity Analysis on DC-DC converter has been done with respect to the variation of parameters of Filtering inductor (L_s_) on system response. Value have been changed from -50% to +50% of their values. Seven testing points are taken into consideration. Performance parameters of Duty Ratio (D) have been chosen. Practically variation in L is within ±10% range. Duty ratio for the buck boost converter [36] can be calculated as:

$$D=\frac{\left(R_{o}+R_{L}\right)}{\left(R_{o}-R_{S}\right)}\left[1-\sqrt{\frac{\left(R_{S}+R_{L}\right)}{\left(R_{o}+R_{L}\right)}}\right]$$ (62)

**Fig 17:**
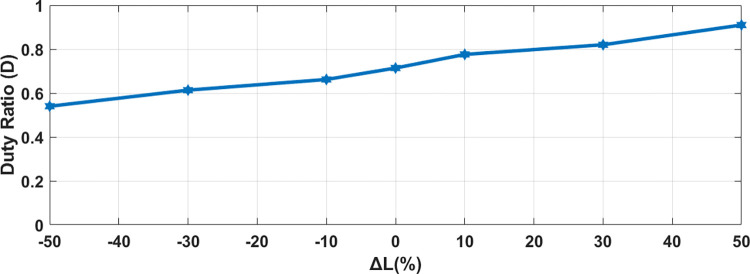
Sensitivity of proposed MPC controller to variations in inductor value for DC-DC converter.

As evident from (62) duty cycle depends on inductor ESR (R_L_). It does not depend on filter capacitor ESR (R_c_). As shown in Fig 17 the variation in filter inductor value do not have considerable effect on duty ratio. These results show that proposed MPC algorithm is less sensitive to parameter change.

## 5. Simulation results

**Fig 18:**
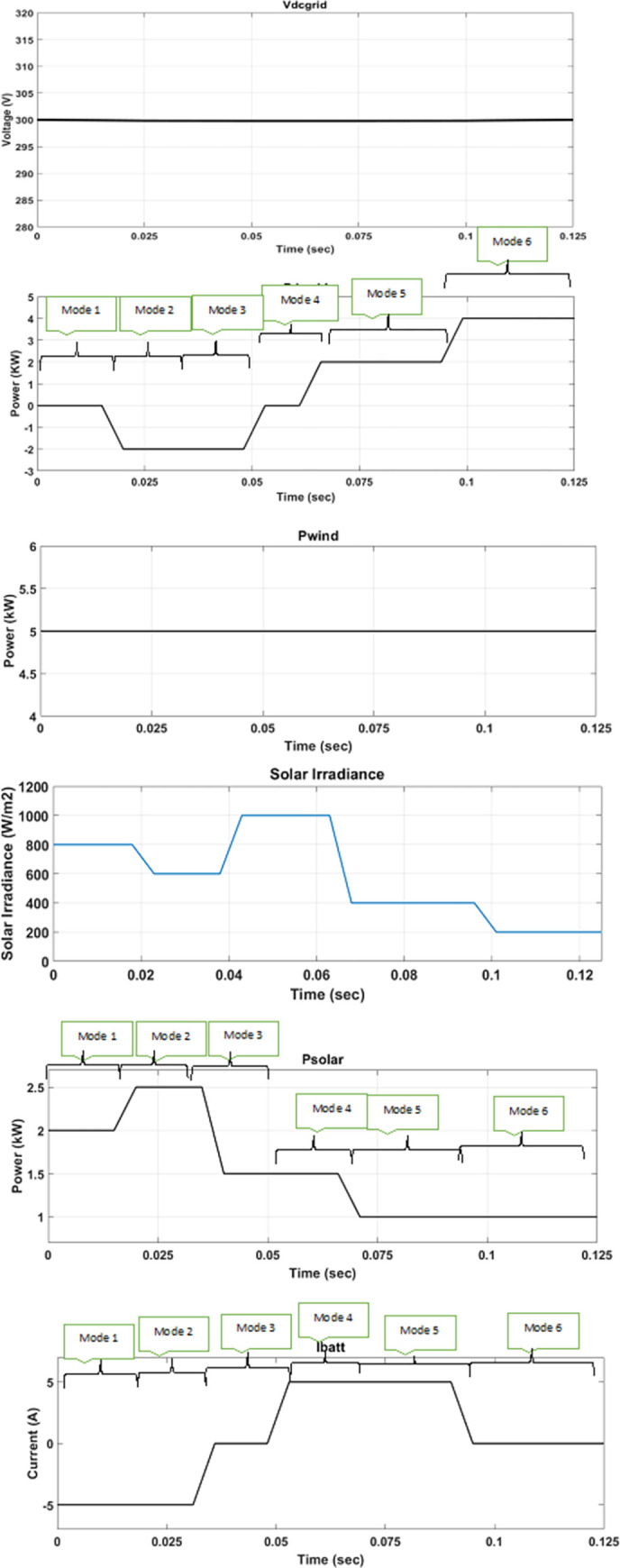
Grid-connected mode under variable DRES generation and load.

**Fig 19:**
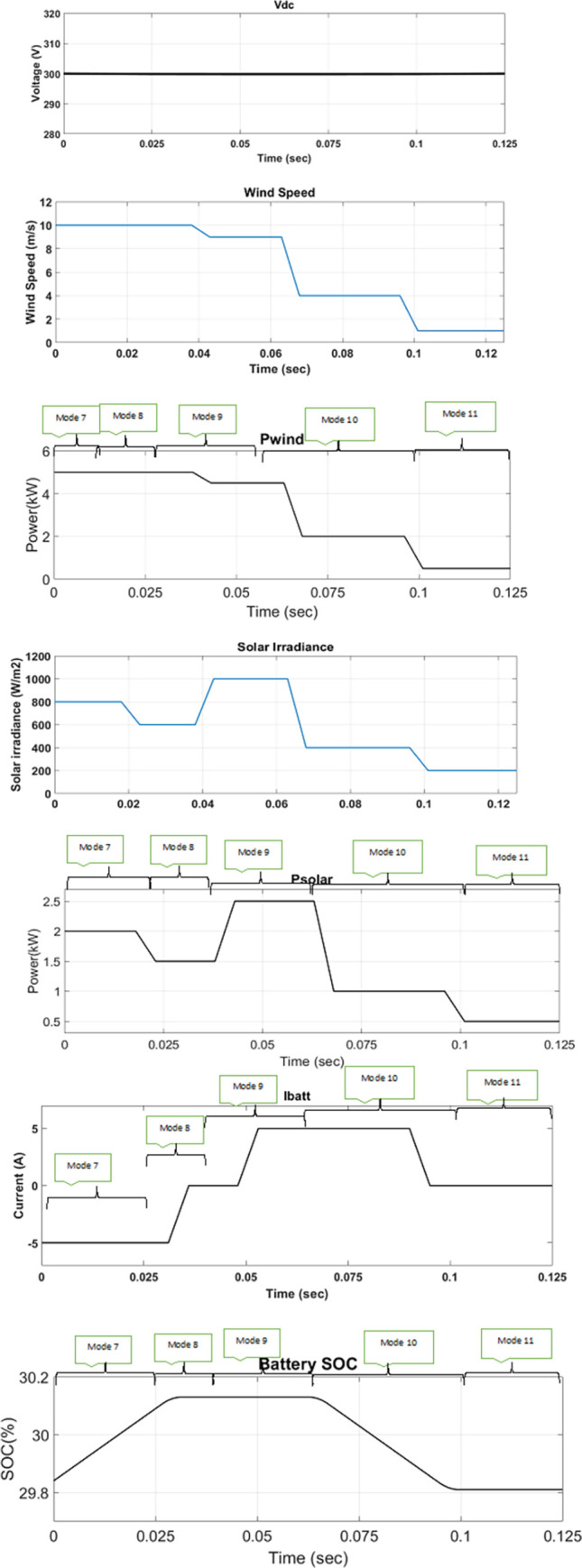
Standalone mode under variable DRES generation and load.

The performance of the proposed MPC algorithm for EMS of the DC microgrid is tested in MATLAB R2019b/Simulink. Parameters used in the simulation are shown in Table 5 in S1 Table attached in the appendix. Loads 1 and 2 are linear loads represented by using constant resistances. Load 3 is modelled as a constant power type in MATLAB/Simulink to represent a non-linear load for purposes of simulation. The proposed MPC algorithm has been compared with PI-based GFM and GFE DGs. Switching frequencies of 20 kHz for the converters have been used to compare both PI and MPC techniques. To verify the efficiency of the proposed method, real-time wind speed and solar irradiation data from Karachi [37] has been used to generate wind and PV output, which are plotted in Figs 18 and 19.

### 5.1. Grid-connected mode

In grid-connected mode, the AC/DC converter acts as GFM DG to maintain DC grid voltage and transfer the power between the DC microgrid and AC microgrid. Wind DG, solar DG and BESS DG act as GFE DG. The BESS operation is determined by the excess power produced and its rated charging/discharging rate as per actual SOC. Different rated loads have been connected to prove the effectiveness of the proposed MPC-based EMS.

Fig 18 shows the simulation waveform for grid-connected mode. At the start, load 1 of 6 kW is connected to the microgrid initially. With the solar irradiance of 800 W/m^2^ and wind speed of 10 m/s, DRES power output is 7 kW. From t = 0 to t = 0.03 s, DRES power output is greater so surplus power is used to charge the BESS, as explained in mode 1 and 2. Moreover, at t = 0.02 s, solar irradiance increases to 1000 W/m^2^, which leads to an increase in solar DG power output. Consequently, excess electricity is here fed back to the AC microgrid, as explained in mode 2. At t = 0.03 s, BESS SOC has reached maximum SOC value (here, it is set at 50.17%), so the BESS no longer requires charging current, and all the excess electricity is fed back to the AC microgrid, as explained in mode 3. At t = 0.03 s, solar DG power output decreases. At t = 0.048 s, total generated power becomes less than load power requirement, so the BESS is discharged to fulfil load power requirement, as explained in mode 4. At t = 0.065 s, solar power output further decreases, and BESS discharge power cannot fulfil the load power requirement. Additional required power is imported from the AC microgrid, as explained in mode 5. At t = 0.09 s, BESS SOC has reached the minimum threshold SOC value (here, it is set at 49.85%), so the BESS discharge current becomes zero. Additional required power is imported from the AC microgrid as explained in mode 6.

### 5.2. Standalone mode

There will be no utility grid in standalone mode, and through the proposed MPC-based EMS, DC microgrid operation will be run smoothly through mode 7 to mode 11. In mode 7 to mode 9, generated power (P_GEN_) is greater than load power (P_Load_), resulting in the BESS being charged. Fig 19 shows the simulation waveform for standalone mode. At the start, load 1 is connected only. With solar irradiance of 800 W/m^2^ and wind speed of 10 m/s, DRES power output is 7 kW. From t = 0 to t = 0.025 s, after fulfilling load 1, surplus power is used to charge the BESS, as explained in mode 7. In mode 7, BESS acts as GFM unit to regulate voltage. At t = 0.025 s, solar power output decreases and total generated power is equal to the load demand, so there will be no power available for BESS charging, as explained in mode 8. In this mode, wind acts as GFM unit while BESS acts as GFE unit. At, t = 0.04 s, wind DG power output decreases and solar power output increases so that total generated power is equal to the load demand and there will be no power available for BESS charging, as explained in mode 9. In this mode, solar acts as GFM unit while wind acts as GFE unit. At t = 0.065 s, total generated power becomes less than the load power requirement due to low solar irradiation and low wind speed, so the BESS is discharged to fulfil load power requirement, as explained in mode 10. In this mode, BESS act as GFM unit to regulate voltage, and solar and wind act as GFE unit to regulate power. At t = 0.1 s, solar and wind power output further decreases, and BESS discharge rated power is less than required load power, so EMS curtail the load equally. BESS acts as GFM unit to regulate voltage, and solar and wind act as GFE unit to regulate power, as explained in mode 11.

**Fig 20:**
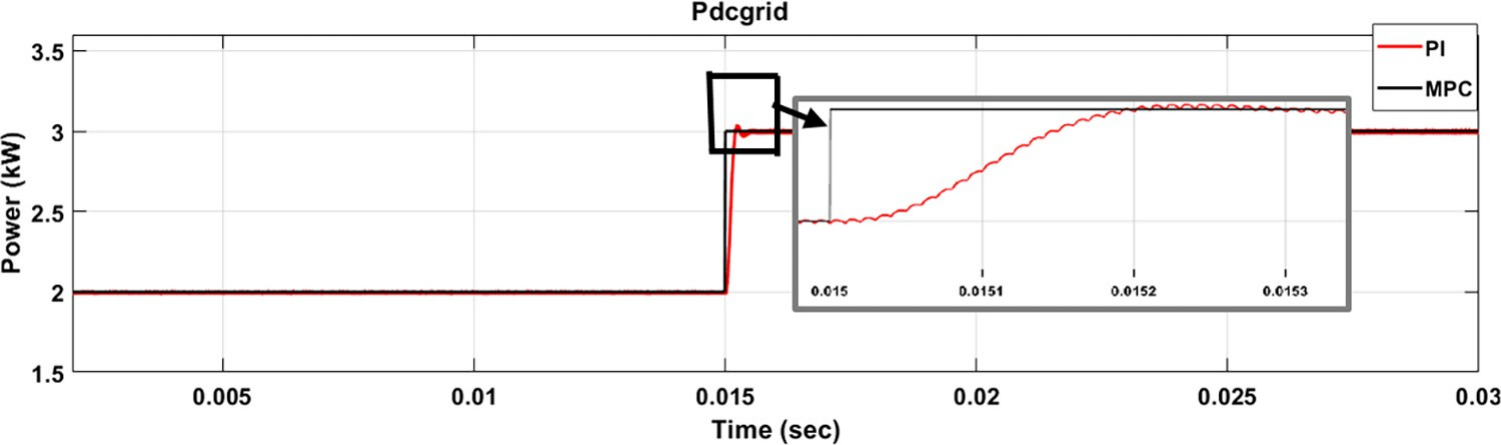
DC bus voltage regulation using PI and MPC.

A comparison of power regulation through MPC and PI is shown in Fig 20. Both MPC and PI-based VSC track the reference power with zero steady-state error. However, the PI controller exhibits undershoot during a transient and larger settling time in comparison to MPC. On the other hand, MPC tracks the reference with faster dynamic response and zero overshoot.

## 6. Conclusions

This paper proposes a consensus-based Energy management system based upon MPC for DRES and BESS individual controllers to operate in both configurations (GFM or GFE). Energy management system determines the mode of power flow based on the amount of generated power, load power, solar irradiance, wind speed, rated power of every DG, and SOC of BESS. Based on selection of power flow mode, the role of DRES and BESS individual controllers to operate as GFM or GFE units is decided. A single MPC hybrid cost function with auto-tuning of weighing factors will enable DRES and BESS converters to switch between GFM and GFE. MPC-based control methods ensure fast mode-changing capability and dynamic response as shown in Table 4. It has been noted that as compared to PI & SMC, MPC technique exhibits settling time of less than 1μsec and 5% overshoot. The results confirm that the proposed MPC-based energy management system ensures the stability of the DC microgrid and local controllers. The results are very encouraging and will play an important part in improving the performance of the energy management system for the DC micro grid using AI techniques with MPC and its testing in hardware in loop setup.

**Table 4:** Comparison of MPC with other techniques.

S. No.	Parameters	PI [38]	SMC [39]	Proposed MPC
1	Rise Time (s)	0.077	0.04	0.03
2	Settling time (s)	0.197	0.0002	0.000001
3	Percentage Overshoot (%)	6.7	9.9	4.2

## Supporting information

S1 Table Simulation parameters. (DOCX)

## Abbreviations

BIC: Bidirectional Interleaving Converter
MPC: Model Predictive Control
DG: Distributed Generation
GFM: Grid Forming
GFI: Grid Following
DRES: Distributed Renewable Energy Sources
SMC: Sliding Mode Control
FCS-MPC: Finite Control Set Model Predictive Contro
PV: Photovoltaic
MG: Microgrid
2L-VSC: Two-Level Voltage Source Converter
BESS: Battery Energy Storage System
GFM DG: GFM Distributed Generation Unit
GFE DG: GFE Distributed Generation Unit
${\vec{v}}_{\mathrm{conv}}$: output voltage
V_dc_: DC bus voltage
$\vec{{\vec{i}}_{error}}(k+1)$: Future value of error vector for the input current
$\vec{v}(k+1)$: Future value of voltage vector
*v*_*sc*_, *v*_*sb*_, and *v*_*sa*_: Three-phase voltages
*i*_*sc*_, *i*_*sb*_, and *i*_*sa*_: Three-phase currents
