# Supplementary material for: Model predictive control of consensus-based energy management system for DC microgrid
Source: PLoS One. 2023 Jan 20;18(1):e0278110. doi: 10.1371/journal.pone.0278110 (PMC9858890; doi:10.1371/journal.pone.0278110)
Supplement: S1 Table — (DOCX) [file pone.0278110.s001.docx]

**Table 5.** Simulation Parameters.

| **Description** | **Values** |
| --- | --- |
| Solar Plant | Trina Solar TSM-250PA05.08, 3 kW. |
| Wind Plant | 5 kW at 10 m/s wind speed (rated) |
| PMSG | 5 kW, L_d_ = L_q_ = 10 mH |
| BESS | 300 V 20 AH |
| DC grid voltage | 300 V |
| AC grid voltage | 110 V |
| Simulation time period | 50 ms |
| Loads | Load 1–6 kW, Load 2–1 kW, Load 3–1 kW. |
